# Divergent LysM effectors contribute to the virulence of *Beauveria bassiana* by evasion of insect immune defenses

**DOI:** 10.1371/journal.ppat.1006604

**Published:** 2017-09-05

**Authors:** Kai Cen, Bing Li, Yuzhen Lu, Siwei Zhang, Chengshu Wang

**Affiliations:** 1 CAS Key Laboratory of Insect Developmental and Evolutionary Biology, Shanghai Institute of Plant Physiology and Ecology, Chinese Academy of Sciences, Shanghai, China; 2 University of Chinese Academy of Sciences, Beijing, China; Wageningen University, NETHERLANDS

## Abstract

The lysin motif (LysM) containing proteins can bind chitin and are ubiquitous in various organisms including fungi. In plant pathogenic fungi, a few LysM proteins have been characterized as effectors to suppress chitin-induced immunity in plant hosts and therefore contribute to fungal virulence. The effector mechanism is still questioned in fungus-animal interactions. In this study, we found that LysM proteins are also present in animal pathogenic fungi and have evolved divergently. The genome of the insect pathogen *Beauveria bassiana* encodes 12 LysM proteins, and the genes were differentially transcribed by the fungus when grown in different conditions. Deletion of six genes that were expressed by the fungus growing in insects revealed that two, *Blys2* and *Blys5*, were required for full fungal virulence. Both proteins could bind chitin and Blys5 (containing two LysM domains) could additionally bind chitosan and cellulose. Truncation analysis of Blys2 (containing five LysM domains) indicated that the combination of LysM domains could determine protein-binding affinity and specificity for different carbohydrates. Relative to the wild-type strain, loss of *Blys2* or *Blys5* could impair fungal propagation in insect hemocoels and lead to the upregulation of antifungal gene in insects. Interestingly, the virulence defects of Δ*Blys2* and Δ*Blys5* could be fully restored by complementation with the Slp1 effector from the rice blast fungus *Magnaporthe oryzae*. In contrast to Slp1 and Blys2, Blys5 could potentially protect fungal hyphae against chitinase hydrolysis. The results of this study not only advance the understanding of LysM protein evolution but also establish the effector mechanism of fungus-animal interactions.

## Author summary

Insect pathogenic fungi are of importance for both applied and basic research. Relative to the advances in understanding fungus-plant interactions, the mechanisms of the molecular pathogenesis of entomopathogenic fungi are rather limitedly understood. In particular, the machinery of effector-mediated inhibition of host immunity has not been well established in fungus-insect interactions. LysM effectors have been characterized as virulence factors in plant pathogens to suppress chitin-triggered immunity in plants. We found that the divergent LysM proteins are also present in animal pathogens. By using the insect pathogen *Beauveria bassiana* as a model, we revealed that two of 12 encoded LysM protein genes *Blys2* and *Blys5* that were transcribed by the fungus growing in insects are required for full fungal virulence against insect hosts. Interestingly, the virulence defects of Δ*Bys2* and Δ*Bys5* could be fully restored by complementation with the divergent Slp1 effector from the plant pathogen *Magnaporthe oryzae*. Both Blys2 and Blys5 can deregulate insect immune responses, and the latter can additionally protect fungal cells from chitinase hydrolysis. The findings of this study establish the contribution of LysM effectors to fungal virulence against insect hosts.

## Introduction

Insect pathogenic fungi such as the ascomycete species *Beauveria bassiana* and *Metarhizium robertsii* have been developed as promising biocontrol agents [1, 2]. Fungal species such as *B*. *bassiana*, *M*. *robertsii* and *M*. *acridum* have also been investigated as genetically tractable systems to unravel the mechanisms of fungus-insect interactions [3]. Similar to plant pathogenic fungi, various strategies ranging from cell wall remodeling to the secretion of immune suppressors have been employed by insect pathogens to evade insect immune responses [3, 4]. For example, the coat protein Mcl1 can be highly expressed by *M*. *robertsii* during fungal growth in the insect hemocoel (body cavity) to camouflage fungal cells from hemocyte recognition and encapsulation [5]. The blastospores of *B*. *bassiana* isolated from insect hemolymph have also been found with shielded carbohydrate epitopes to counteract insect immune defenses [6, 7]. It has also been reported that small molecules such as the cyclopeptide destruxins produced by *Metarhizium* species and the red pigment oosporein produced by *B*. *bassiana* can facilitate fungal infection of insect hosts by inhibiting host immunities [8, 9]. Relative to the well-understood mechanisms of fungus-plant interactions [10], the effector-mediated perturbation of host immunities has not been fully elucidated in animal pathogens including both insect and mammalian pathogenic fungi.

The chitin in cell walls is a well-characterized pathogen-associated molecular pattern (PAMP) in plant pathogenic fungi that can trigger host immune responses [11]. The lysin motif (LysM)-containing receptor kinases have been investigated in plants for mediating recognition of PAMP chitin during microbe-plant interactions [12, 13]. The diverse LysM proteins are also widely distributed in the fungal kingdom and have been characterized as chitin-binding effectors in plant pathogens to deregulate host immunity [14]. Of these, Ecp6 is the first characterized LysM effector that contributes to the virulence of the tomato leaf mold *Cladosporum fulvum* [15, 16]. It was later found that LysM proteins can also function as virulence factors in other plant pathogens, such as Slp1 in the rice blast fungus *Magnaporthe oryzae* [17], Mg1LysM and Mg3LysM in the wheat pathogen *Mycosphaerella graminicola* [18], ChElp1 and ChElp2 in the plant anthracnose fungus *Collectotrichum higginsianum* [19], and Vd2LysM in the soil borne wilt disease *Verticillium dahliae* [20]. The virulence effect of LysM proteins in animal pathogenic fungi is still unclear.

Functional studies of Ecp6 and Slp1 revealed that these LysM effectors could sequester the chitin oligosaccharides released from the cell walls of invading fungal hyphae to prevent the activation of plant immunity and/or outcompete the host immune receptor for chitin binding [16, 17, 21]. The chitin in fungal cell walls can be targeted and degraded by plant chitinases [12]. LysM effectors, such as the Mg1LysM and Mg3LysM of *M*. *graminicola*, can protect fungal hyphae against the hydrolytic activity of plant-derived chitinases [22]. In contrast, both Slp1 and Ecp6 cannot protect fungal cells from chitinase hydrolysis [16, 17]. LysM proteins are also widely distributed in animal fungal pathogens [23, 24], and chitin can trigger immune responses in both mammals and insects [25]. These data would suggest the existence of a LysM effector machinery in fungus-animal interactions, which, however, has been questioned due to the consideration of the non-intimate relationships between fungi and animals compared to that which exist for plasmalemma-enveloped fungus and plant cells [11, 26]. Molecular evidence is still required to substantiate these arguments.

Our previous genomic analysis of insect pathogenic fungi identified an array of plant pathogen-like effectors, including LysM proteins [24, 27]. In this study, we characterized the function of LysM proteins encoded in the genome of the insect pathogenic fungus *B*. *bassiana* and found that divergent proteins can bind chitin polymers and fungal cell walls to deregulate insect immune defenses. Of particular interest, we found that the Slp1 effector from the rice blast fungus could restore the virulence defect of the gene deletion mutants against insect hosts.

## Results

### Divergent evolution of LysM proteins between and within fungal lineages

**Fig 1:**
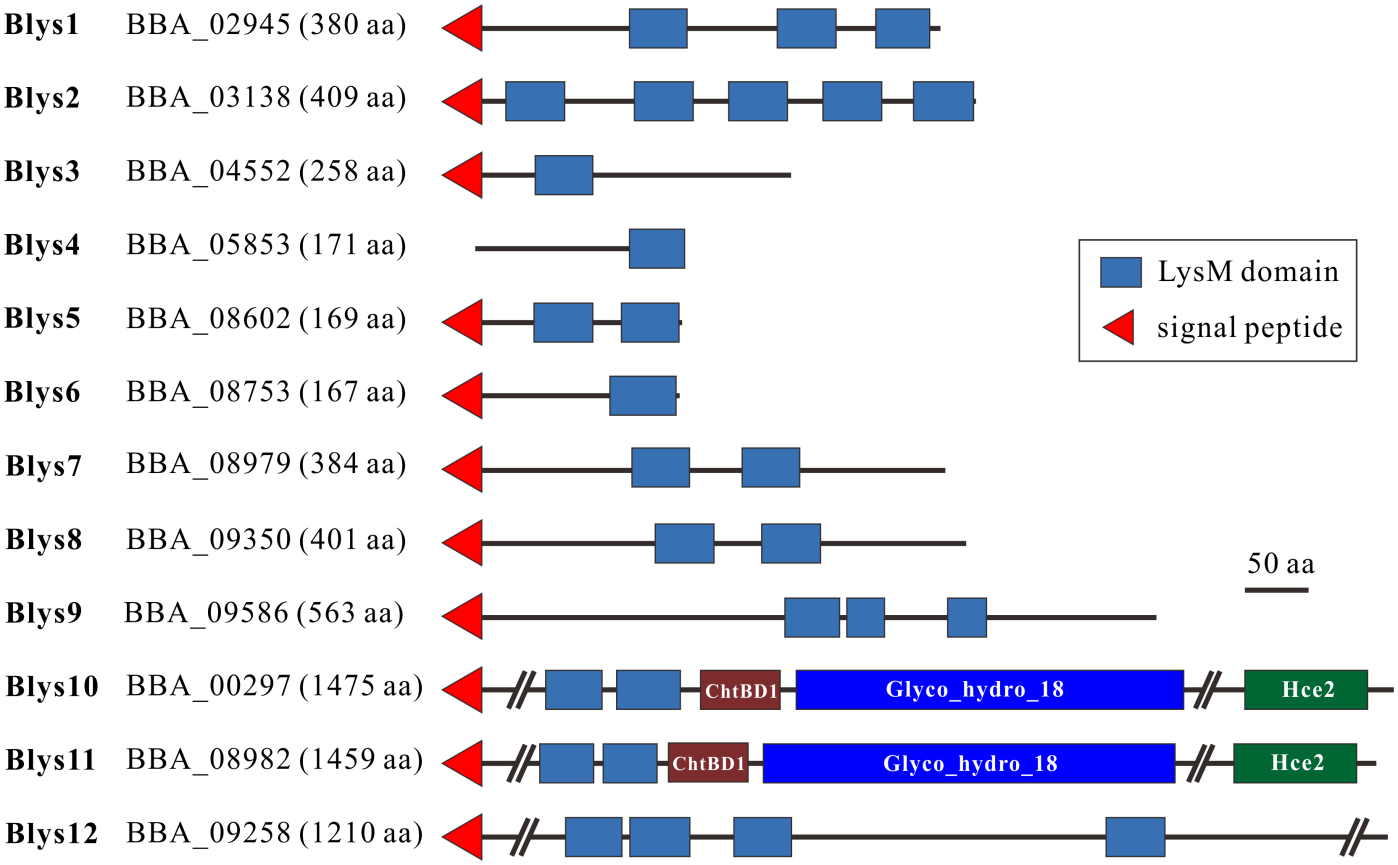
Structure modulation of the LysM proteins encoded by *B*. *bassiana*. ChtBD1, chitin-binding domain; Glyco_18, GH18 chitinase domain; Hce2, homolog of *Cladopsorium fulvum* Ecp2 effector domain.

We first performed bioinformatic analyses of fungal LysM proteins by including those from insect and mammalian pathogenic fungi. Consistent with previous observations [14, 28], we found that the number of LysM proteins varied highly among fungal species (S1 and S2 Tables). For example, 12 LysM proteins are present in the genome of *B*. *bassiana* (termed Blys1-Blys12, Fig 1) whereas 13 in *Metarhizium robertsii*, eight are present in *M*. *oryzae* and 18 in *C*. *higginsianum*. Fungal LysM proteins are usually cysteine-rich and vary in length [14]. By plotting protein length versus the cysteine ratio for each protein, we found that the 282 examined fungal proteins could be divided into two groups: one group containing proteins of 90–850 aa and 2–7% cysteine residues (g1 group), and the other group containing proteins > 850 aa and 2.5–4% cysteine (g2 group) (S1 Fig). In the g2 group, the proteins from the plant pathogens are highly underrepresented (5/96) compared to those from the insect (41/133) and mammalian (10/53) pathogens. In addition, we found that most LysM proteins contain a signal peptide (78% of 133 examined proteins from insect pathogens) (S1 Table), which is considerably higher than the genome-wide average (ca. 15%) for secretable proteins [29].

As evident in 12 LysM proteins from *B*. *bassiana* (Fig 1), varied numbers (1–7) of LysM domains (termed as LysMs for abbreviation) are present in various fungal proteins, which may or may not contain the additional ChtBD1 type of chitin-binding domain, the Glyco_18 type of chitinase domain and/or the Hce2 effector domain (Pfam: PF14856) (S2 Fig). Phylogenetic analysis indicated that the examined proteins could be grouped into different lineages partially through association with protein structures. For example, most of the large LysM proteins that contain the ChtBD1, Glyco_18 and/or Hce2 domains are clustered together (Cluster I), as are the intracellular proteins with a single LysM domain (Cluster II, including Blys4) (S2 Fig). For Blys1-12 of *B*. *bassiana*, Blys10 and Blys11, and Blys9 and Blys12 are close to each other, whereas the rest of the Blys proteins fall into different lineages. Individual LysM sequences were also retrieved from each protein for phylogenetic analysis, and the results indicated that more than five clustering patterns could be obtained (S3 Fig). Even most of LysMs were clustered together independent of their sequential positions within the parental proteins (C4 cluster), the LysM1 (C2 cluster) and LysM2 (C3 cluster) from those proteins containing additional ChtBD1 and Glyco_18 domains could be grouped into respective lineages (S3 Fig), i.e., the relationships with protein structures.

The LysMs of the effectors Ecp6, Slp1 and Mg3LysM are more similar to the counterparts from bacteria with zero or one cysteine residue [14]. Consistently, the LysM domains from these proteins were clustered together into a basal lineage (S3 Fig). In addition, further analysis of LysM sequence consensus indicated that the LysMs from plant pathogens could be divided into two types, i.e., the pattern similar to those from bacteria (S4A and S4B Fig), and the pattern containing four cysteine residues (S4C Fig). The LysMs from insect pathogens contain four cysteine residues (S4D Fig), i.e., the typical fungal-specific LysMs that can putatively form two disulfide bridges within each domain [14]. The divergently evolved LysM proteins suggest functional diversities of these proteins in fungal biology.

### Differential gene expressions

**Fig 2:**
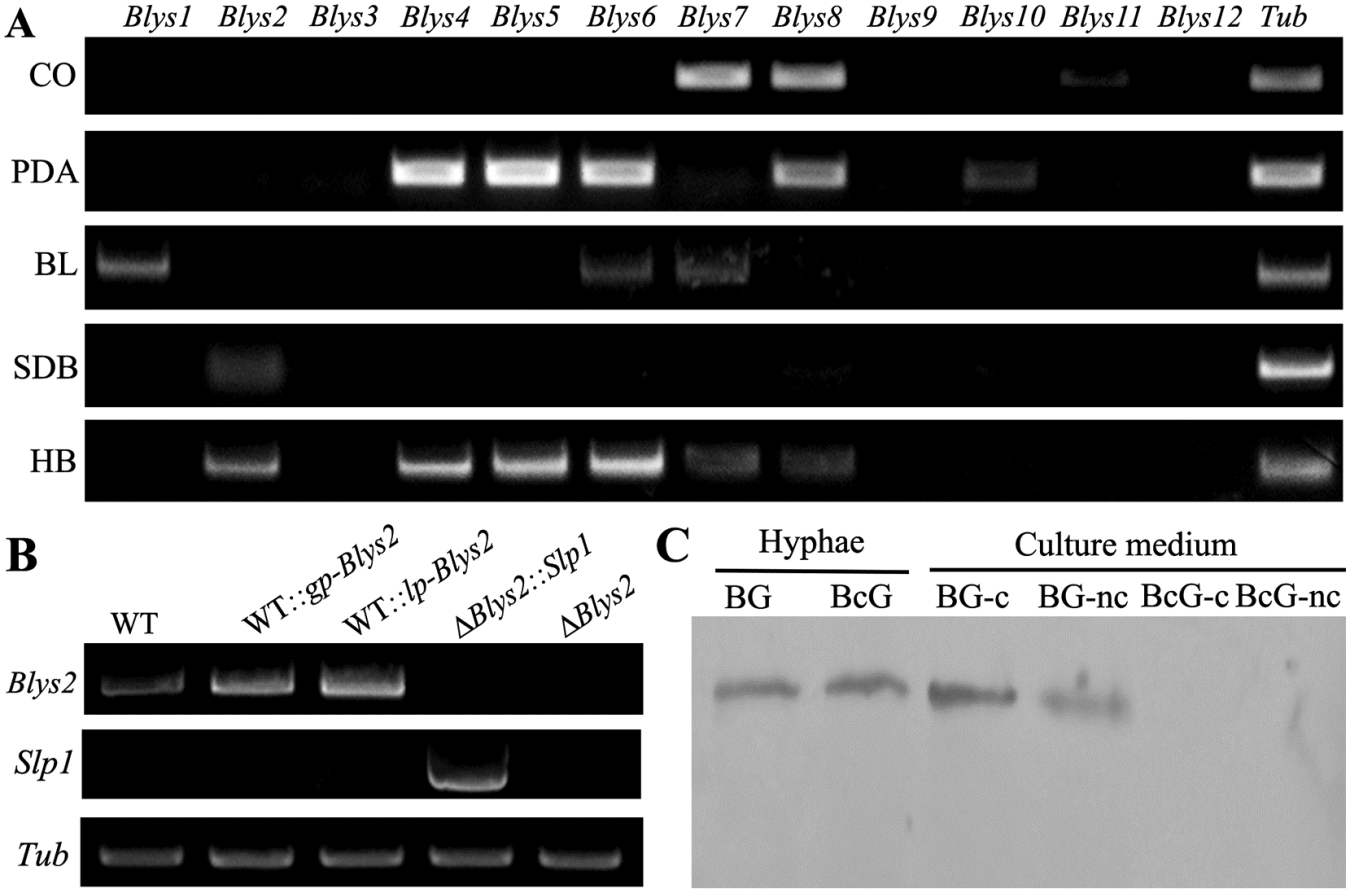
Gene and protein expression assays. **A**. RT-PCR analysis of the expression of 12 putative LysM protein genes in *B*. *bassiana* after growth in different nutrients. CO, conidia harvested from PDA for ten days; PDA, mycelium sample harvested from the PDA medium for three days; BL, blastospores harvested from the SDB culture for seven days; SDB, mycelia harvested from the SDB for three days; HB, hyphal bodies harvested from insect hemolymph 36 hrs post injection of fungal spores. Tub, β-tubulin gene used as a reference. **B**. RT-PCR verification of gene deletion and genetic engineering for overexpression. WT and mutants were grown in SDB for three days and cultures were harvested for RNA extraction and gene expression assay. **C**. Western blotting verification of Blys2 secretion feature. BG, Blys2-GFP, Blys2 fused in frame with GFP; BcG, Blys2-SP-GFP, Blys2 without signal peptide in fusion with GFP protein; nc, non-concentrated; c, concentrated. The anti-GFP antibody was used for analysis.

As indicated above, 12 LysM proteins are encoded by *B*. *bassiana*, and these proteins vary in length and contain a different number of LysM and/or other domains (Fig 1). Except for Blys4, all other proteins each contain a signal peptide. We performed gene expression analysis by growing the fungus in various conditions, including the *in vivo* infection stages within the insect hemocoels. The results indicated that these genes were differentially expressed by the fungus (Fig 2A). For example, *Blys7* and *Blys8* were highly transcribed in the conidia. Relative to growth on solid medium, fewer genes were expressed by the fungus growing in an artificial liquid medium. In particular, six genes (i.e., *Blys2*, *Blys4*, *Blys5*, *Blys6*, *Blys7* and *Blys8*) were differentially transcribed by the fungus during *in vivo* infection of insect hosts. The closely related *Blys10* and *Blys11*, and *Blys9* and *Blys12* remained largely silent in *B*. *bassiana* under the examined conditions.

### The requirement of Blys2 and Blys5 for fungal virulence against insects

The LysM effectors expressed by plant pathogens during colonization of hosts are required for fungal virulence [11]. To examine the virulence contribution of LysM proteins in *B*. *bassiana*, we performed homologous recombination-mediated deletions of the six genes upregulated by the fungus growing in insects. Different null mutants were obtained and verified by reverse-transcription PCR (RT-PCR) (S5A Fig). Deletion of these genes had no obvious negative effect on fungal growth on potato dextrose agar (PDA) and PDA amended with Calcofluor White. However, Δ*Blys4* and Δ*Blys5* became relatively tolerant against H_2_O_2_-induced oxidative stress when compared to the wild type (WT) (S5B Fig). Both the WT and mutant cultures could not grow at 37°C. We conducted both injection and topical infection bioassays using the last instar larvae of the wax moth *Galleria mellonella* to compare the virulence difference between the WT and null mutants of *B*. *bassiana*. The estimation and statistical comparison of the median lethal time (LT_50_) values indicated that the deletions of *Blys2* and *Blys5*, but not the other genes, significantly (*P* < 0.01) impaired fungal virulence in both types of bioassays (Table 1; S6 Fig). For example, during the injection assays, the LT_50_ values of Δ*Blys2* (3.133 ± 0.095 d) and Δ*Blys5* (2.944 ± 0.108 d) was significantly (*P* < 0.01) extended compared to that of the WT (2.600 ± 0.097 d). Similar to the virulence contributions of the LysM effectors in plant pathogens [11, 28], both Blys2 and Blys5 are therefore required for the full virulence of *B*. *bassiana* to infect insect hosts. Unfortunately, the trials to delete both the *Blys2* and *Blys5* genes were not successful for reasons that remain unclear.

**Table 1:** Comparison of the virulence between the WT and mutants assayed against the wax moth larvae.

Strains	Injection		Topical infection	
	LT_50_ (days)	Significance*	LT_50_ (days)	Significance*
WT	2.600 ± 0.097	—	4.322 ± 0.172	—
Δ*Blys2*	3.133 ± 0.095	χ^2^ = 13.381; *P* = 0	5.156 ± 0.142	χ^2^ = 8.610; *P* = 0.003
Δ*Blys4*	2.589 ± 0.097	χ^2^ = 0.03; *P* = 0.957	4.556 ± 0.174	χ^2^ = 0.655; *P* = 0.418
Δ*Blys5*	2.944 ± 0.108	χ^2^ = 8.987; *P* = 0.003	5.089 ± 0.166	χ^2^ = 8.272; *P* = 0.004
Δ*Blys6*	2.633 ± 0.093	χ^2^ = 0.074; *P* = 0.785	4.522 ± 0.189	χ^2^ = 1.196; *P* = 0.274
Δ*Blys7*	2.500 ± 0.088	χ^2^ = 0.643; *P* = 0.423	4.467 ± 0.168	χ^2^ = 0.255; *P* = 0.255
Δ*Blys8*	2.711 ± 0.097	χ^2^ = 0.738; *P* = 0.390	4.322 ± 0.172	χ^2^ = 0.795; *P* = 0.373
WT::*gp-Blys2*	2.522 ± 0.112	χ^2^ = 1.321; *P* = 0.251	4.500 ± 0.162	χ^2^ = 0.102; *P* = 0.749
WT::*lp-Blys2*	2.222 ± 0.091	χ^2^ = 2.094; *P* = 0.148	4.589 ± 0.141	χ^2^ = 0.277; *P* = 0.599
Δ*Blys2*::*Slp1*	2.211 ± 0.093	χ^2^ = 2.017; *P* = 0.156	4.611 ± 0.149	χ^2^ = 0.563; *P* = 0.453

*, The significance of difference was estimated between the WT and individual mutant by the Log-rank test. Each strain had three replicates with 15 insects each, and the experiments were repeated twice.

Additional mutants were generated to overexpress *Blys2* in the WT strain, whereby *Blys2* expression was made under the control of the constitutive promoter of either the *GpdA* or laccase gene of *B*. *bassiana* [30]. We also tried to rescue the *Blys2* and *Blys5* deletion mutants using the *M*. *oryzae Slp1* gene under the control of the *GpdA* promoter for transformation. Both *Blys2* and *Slp1* could be successfully transcribed by the obtained mutants (Fig 2B). The injection and topical infection bioassays indicated that the LT_50_ values of *Blys2* overexpression mutants had no obvious difference (*P* > 0.1) from those of the WT. Interestingly, complementation of Δ*Blys2* with *Slp1* fully restored the virulence defect of Δ*Blys2* (Table 1; S7A and S7B Fig). Likewise, the statistical difference between WT and Δ*Blys5*::*Slp1* was non-significant (χ^2^ = 2.081; *P* = 0.149) (S7C Fig). Nevertheless, the full sequence and two LysM domains of Slp1 exhibited no obvious conservation and phylogenetic relatedness with those of Blys2 and Blys5 (S8 Fig).

### Chitin binding and cell wall protection assays

**Fig 3:**
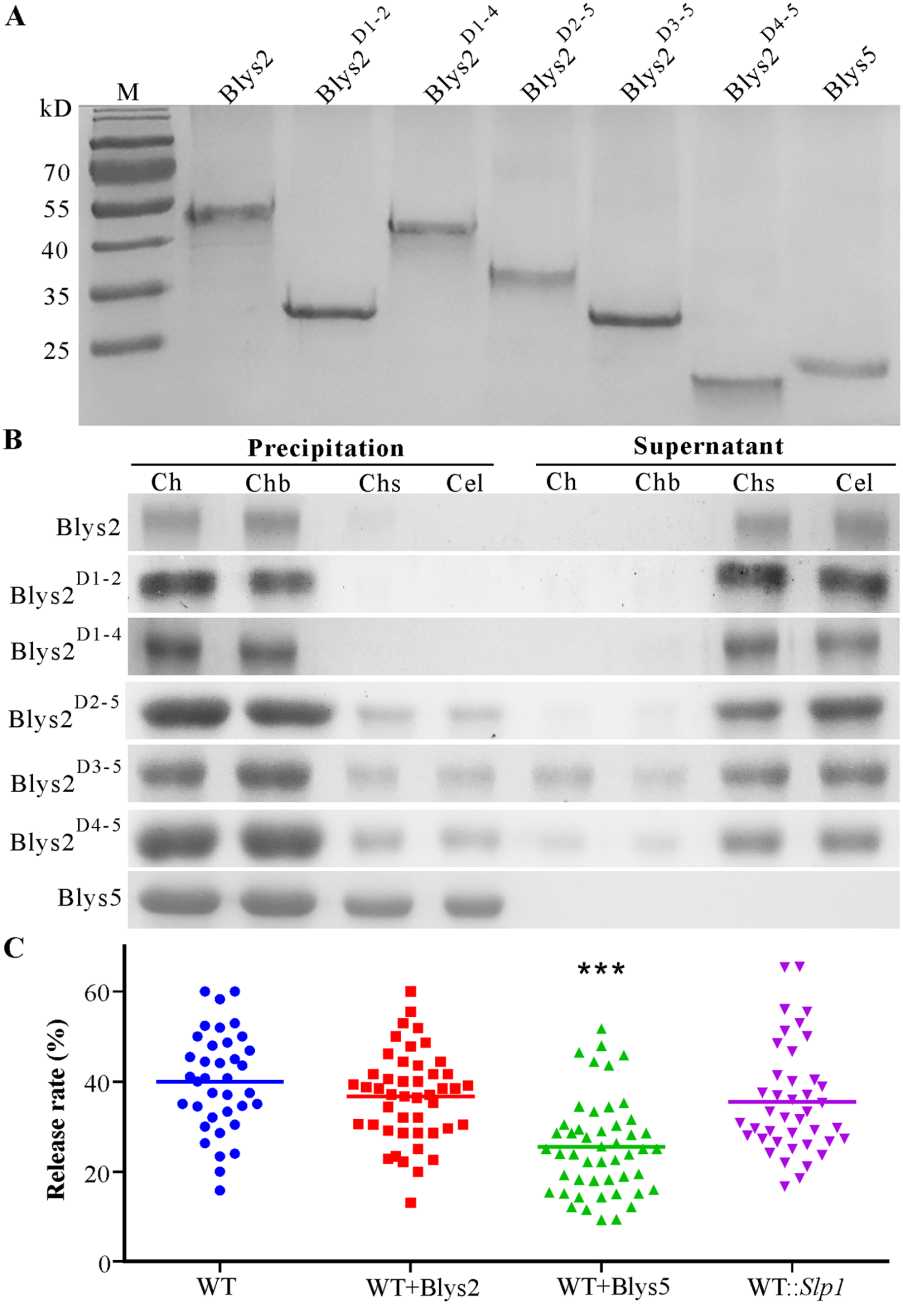
Protein solubility, polysaccharide binding and cell wall protection assays. **A**. Polyacrylamide gel analysis of protein solubility. The protein samples were centrifuged at a maximum speed for 5 min and aliquots of each sample (10 μg each) were analyzed on a 12% SDS-PAGE gel. M, protein marker. **B**. Protein binding assay. After incubation of WT Blys2, truncated forms of Blys2 and Blys5 proteins with various polysaccharides, the supernatant and polysaccharide precipitated samples were run through a 12% SDS-PAGE gel and stained with the Coomassie brilliant blue to examine the protein-binding features. Ch, chitin extracted from fungal walls; Chb, chitin beads; Chs, chitosan; Cel, cellulose. The truncated forms of Blys2 are indicated as, e.g., Blys2^D1-2^ for the truncated Blys2 only containing the first two LysM domains and Blys2^D1-4^ for the truncated Blys2 only containing the 1–4 LysM domains etc. **C**. Cell wall protection from chitinase hydrolysis. The germlings were pre-incubated with either Blys2 or Blys5 for 2 hrs before the treatment with a chitinase cocktail for 2 hrs. The release rate of protoplast was quantified and compared. The WT and WT::*Slp1* germlings without the pre-incubation with either protein were treated with chitinase cocktails as controls. ***, *P* < 0.0001.

It has been found that the LysM domains determine the protein-binding affinity to chitin or specificity to other type of carbohydrates [14]. Blys2 contains five LysMs whereas Blys5 has two (Fig 1). To determine their binding abilities to different carbohydrates, the cDNAs of both genes were cloned into the vector for expression in *Escherichia coli*. In addition, different truncated forms of Blys2 with a reduced number of LysM domains were also expressed. The purified proteins were all soluble (Fig 3A). The saturated affinity binding assays were performed using the chitin polymers extracted from the conidial cell wall of *B*. *bassiana*, chitin beads, chitosan and cellulose. The results indicated that, similar to Ecp6 [16] and Slp1 [17], both Blys2 and Blys5 could bind fungal cell wall chitin and chitin beads. However, in contrast to Blys2, Blys5 could also target chitosan and cellulose (Fig 3B). The truncated forms of Blys2, i.e., Blys2^D1-2^ (Blys2 only contains first two LysM domains), Blys2^D1-4^, Blys2^D2-5^, Blys2^D3-5^ and Blys2^D4-5^, could also bind fungal chitin and chitin beads. Unlike Blys2^D1-2^ and Blys2^D1-4^, the other forms also slightly bound chitosan and cellulose. In addition, relative to the full protein and other truncated forms, the binding ability of the forms Blys2^D3-5^ and Blys2^D4-5^ was reduced, as these truncated proteins were also detected in the supernatant samples (Fig 3B).

Different LysM proteins play a distinct role in protecting fungal cells from chitinase hydrolysis [17, 22]. To determine the protection potential of Blys2 and Blys5 against chitinase, the WT spores were germinated in a liquid medium for 16 hrs. The germlings were incubated with each protein and then treated with chitinase cocktails to compare the difference in formation of protoplasts. The results indicated that a similar ratio (*P* = 0.1618) of protoplasts was released from the Blys2-treated sample whereas a significantly fewer number of protoplasts was formed from the Blys5-incubated germlings (*P* < 0.0001) when compared to the mock control (the WT germlings without protein incubation). Consistent with the previous report that Slp1 cannot protect fungal hyphae from chitinase degradation [16], non-significant difference (*P* = 0.0919) was observed between the mock and WT::*Slp1* samples (Fig 3C). Thus, Blys5 but not Blys2 can protect fungal hyphae against the hydrolytic activity of chitinase.

### Fungal cell wall binding by Blys2

A recent report indicated that Blys2 was identified as a cell wall protein from both the blastospores and hyphal bodies of *B*. *bassiana* [7]. To determine the secretion and localization feature of Blys2, a green fluorescent protein (GFP) was fused in frame at the C-terminus of Blys2. The cleaved form of Blys2 without the signal peptide (Blys2-SP) was also generated, and both cassettes were placed under the control of the *GpdA* gene promoter for transformations of the WT strain of *B*. *bassiana*. Both types of proteins could be successfully expressed and detected in the mycelial-protein samples of *B*. *bassiana* by Western blot analysis using an antibody against the GFP protein. However, in contrast to the WT Blys2, Blys2-SP could not be detected in the culture medium. The result confirmed that Blys2 is an extracellular protein (Fig 2C).

**Fig 4:**
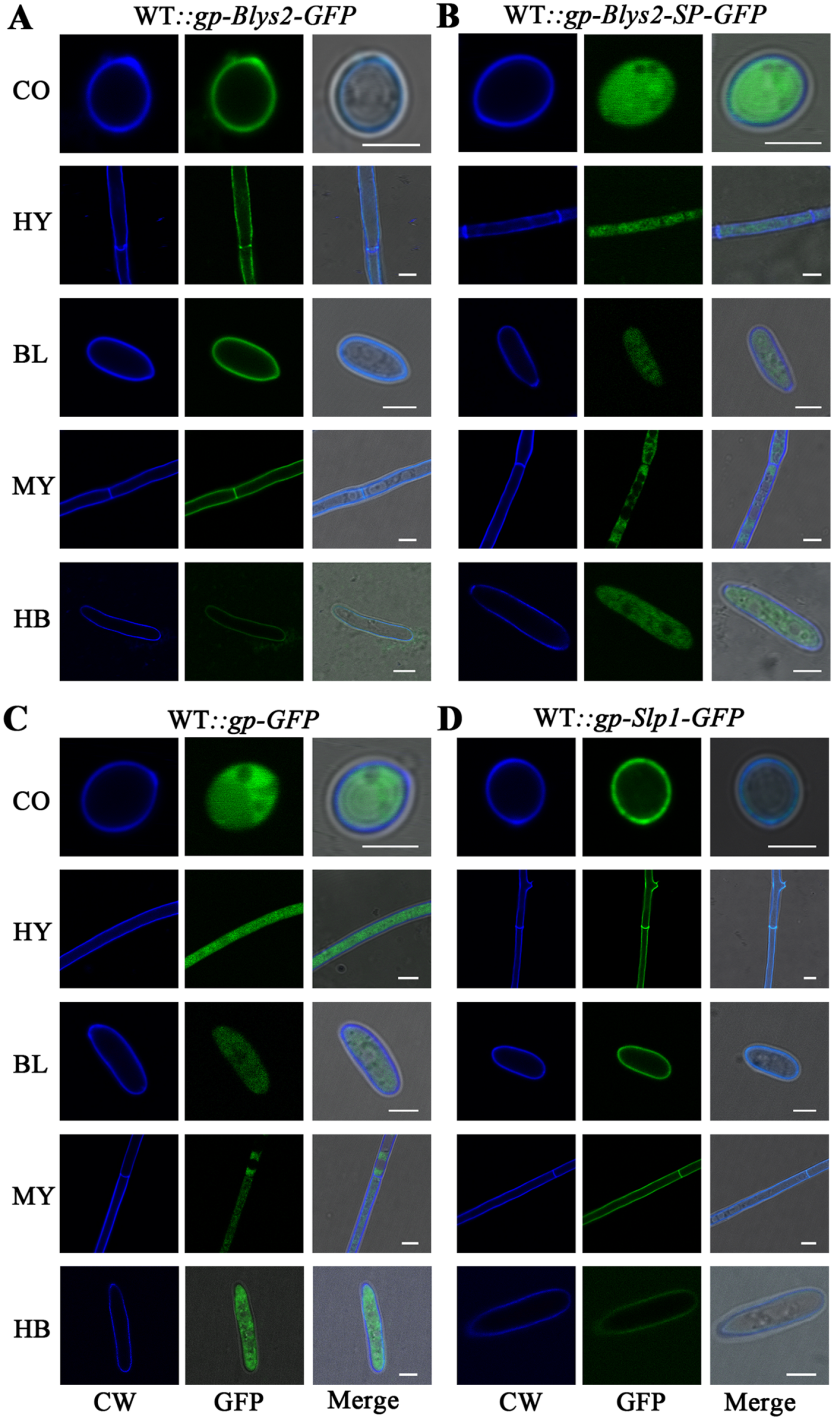
Protein localization assays. **A**. Localization of Blys2 on the fungal cell walls of different type cells. Full length *Blys2* was fused in frame with a *GFP* gene and the cassette was controlled by the *GpdA* gene promoter for transformation of the WT strain of *B*. *bassiana*. **B**. Cytosolic localization of Blys2 without signal peptide (Blys2-SP) in different cells. The truncated *Blys2* without signal peptide sequence was fused in frame with a *GFP* gene and the cassette was controlled by the *GpdA* gene promoter for transformation of the WT strain of *B*. *bassiana*. **C**. Cytosolic localization of GFP protein. The *GFP* gene was controlled by the *GpdA* gene promoter for transformation of the WT strain of *B*. *bassiana*. **D**. Localization of Slp1 on the cells walls of *B*. *bassiana* cells. Full length *Slp1* of *M*. *oryzae* was fused in frame with a *GFP* gene and the cassette was controlled by the *GpdA* gene promoter for transformation of the WT strain of *B*. *bassiana*. The cells of different mutants were examined and photographed under the 100 × field of a confocal microscope. CO, conidia; HY, hyphae harvested from the PDA plate for three days; BL, blastospore; MY, mycelia harvested from SDB for three days; HB, hyphal body harvested from insect hemocoels. CW, Calcofluor White. Bar, 2 μm.

To examine the localization of Blys2, fungal cells were stained with the fluorochrome Calcofluor White for chitin labeling. We found that the Blys2-GFP signal could be detected in the chitin-stained cell walls of various cell types (labelled as WT::*gp-Blys2-GFP*), including conidial spores, hyphae, blastospores and hyphal bodies (Fig 4A). This result was consistent with the observation that Blys2 can bind fungal cell wall chitin (Fig 3B). By contrast, smeared GFP signals were observed in mutant cells (labelled as WT::*gp-Blys2-SP-GFP*) expressing Blys2-SP-GFP (Fig 4B). This finding was similar to those obtained from the cells (labelled as WT::*gp-GFP*) that were only transformed with a *GFP* gene (Fig 4C). In addition, consistent with the chitin-binding nature of Slp1 [16], we found that GFP-fused Slp1 could also be detected in the mutant (labelled as WT::*gp-Slp1-GFP*) cell walls of *B*. *bassiana* (Fig 4D). In comparison to other types of cells, chitin-staining, Blys2-GFP and Slp1-GFP signals were more weakly detected in hyphal bodies that were harvested from insect hemocoels. This result could be due to the occurrence of cell wall re-modification with reduced contents of chitin and β-glucans during fungal growth in insect body cavities [31].

### Evasion of insect immunities

**Fig 5:**
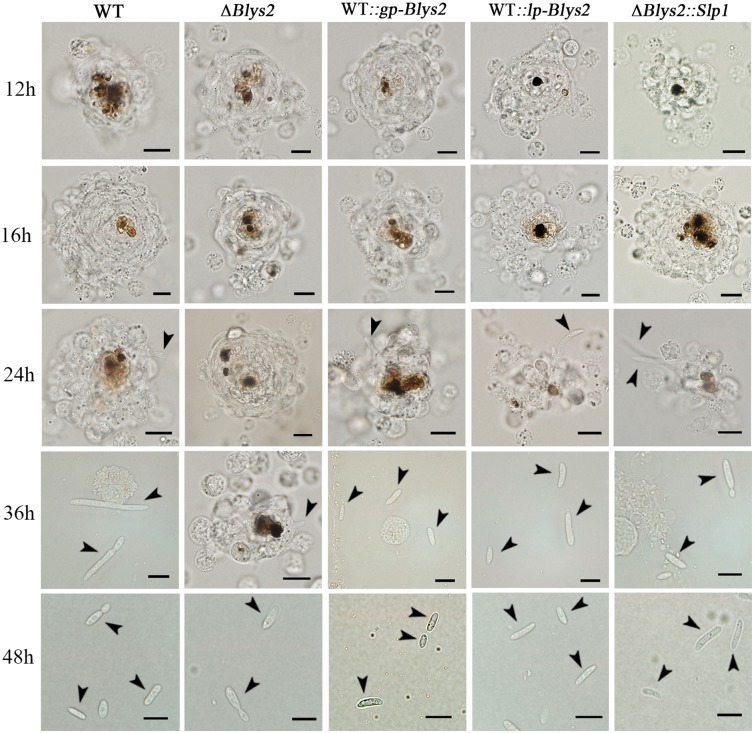
Insect hemocyte encapsulation and fungal developments in insect hemocoels on a time scale. The last instar larvae of wax moth were injected with spores of WT and mutants and bled at different times (labeled on the left) for microscopic examination of insect cellular immune responses and fungal developments. Bar: 10 μm. Arrows in different panels point to fungal cells. For overexpression of *Blys2* in the WT strains of *B*. *bassiana*, both the *GpdA* (to obtain the transformant WT::*gp-Blys2*) and laccase (WT::*lp-Blys2*) gene promoters were used to control gene transcription.

**Fig 6:**
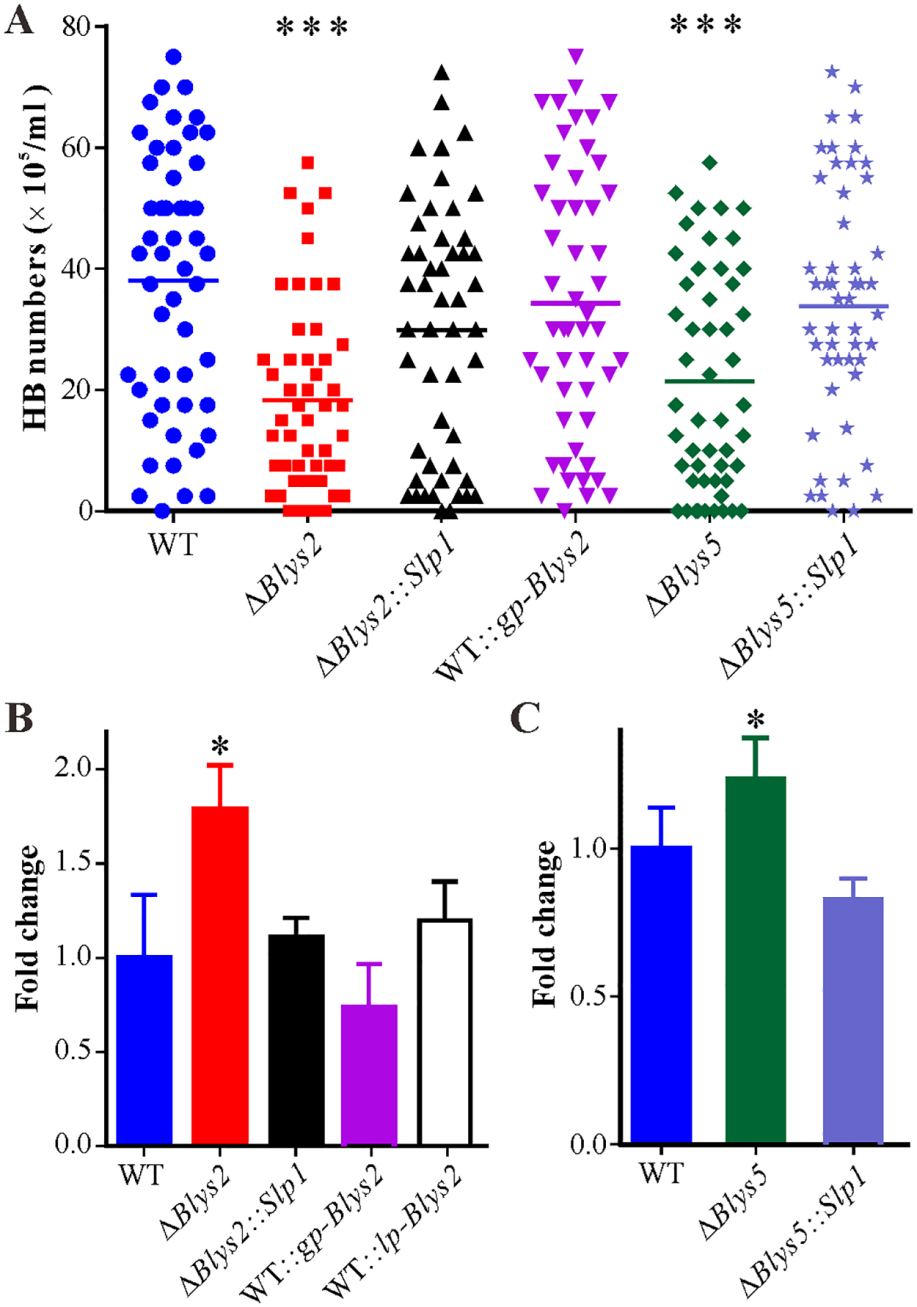
Comparison of fungal cell propagation and antifungal gene expression in insects. **A**. Comparison of fungal hyphal body (HB) numbers in insect hemocoel 48 hrs post injection. Ten insects were bled for each treatment, and five microscopic fields were observed for each insect. Unpaired *t*-test was conducted to compare the difference level between WT and mutants. ***, represents the difference level at *P* < 0.001. **B**. qRT-PCR analysis of *Gal* (for gallerimycin) gene expression by insects treated with spores of WT and *Blys2*-related mutants for 36 hrs. *, *P* < 0.05. **C**. qRT-PCR analysis of *Gal* gene expression by insects treated with spores of WT and *Blys5*-related mutants for 36 hrs. *, *P* < 0.05. The *Slp1* gene of *M*. *oryzae* was used to complement the null mutants of *Blys2* (Δ*Blys2*::*Slp1*) and *Blys5* (Δ*Blys5*::*Slp1*), respectively. Overexpress of *Blys2* in the WT strain of *B*. *bassiana* was performed by using two different constitutive promoters, i.e., the *GpdA* (to obtain the transformant WT::*gp-Blys2*) and laccase gene (to obtain the transformant WT::*lp-Blys2*) promoters.

Having established that Blys2 and Blys5 are required for full fungal virulence and can bind fungal cell wall chitin, we performed further experiments to investigate the mechanism of protein virulence contribution. Thus, the spores of the WT, Δ*Blys2*, Δ*Blys5* and *Slp1*-rescued mutants were injected into the last instar of wax moth larvae, and the insects were bled at various times to examine fungal developments. We found that the typical cellular immune responses, i.e., hemocyte encapsulation and melanization [5], similarly occurred in insects against both WT and mutant spores up to 24 hrs post injection (Fig 5). However, in contrast to Δ*Blys2*, the WT, *Blys2* overexpression (i.e., the mutants WT::*gp-Blys2* and WT::*lp-Blys2*) and *Slp1*-rescued mutants (Δ*Blys2*::*Slp1*) began to produce hyphal bodies 36 hrs post treatments. Subsequently, significantly fewer (*P* < 0.0001) free-living cells were produced by Δ*Blys2* when compared to the WT 48 hrs post injection. We also found that, relative to the WT, the propagation of Δ*Blys5* was considerably (*P* < 0.0001) impaired in insect hemocoels as well. However, no obvious differences (*P* > 0.05) were observed between the WT and other mutants (Fig 6A). In addition, a quantitative real-time PCR (qRT-PCR) analysis indicated that the expression of the antifungal gallerimycin gene could be more highly (*P* < 0.05) induced in insects infected by Δ*Blys*2 than by the WT (Fig 6B). In comparison to the WT, deletion of *Blys5* could also lead to a higher level (*P* < 0.05) of induction of the antifungal gene expression in insects (Fig 6C). However, no significant difference was observed between the WT and other mutants including the *Slp1*-rescued mutants. Thus, deletion of either *Blys2* or *Blys5* could impair fungal propagations and ability in suppressing immune responses in insects, and the mutant defects could be complemented with the *Magnaporthe Slp1* gene.

## Discussions

To perturb chitin detection by host cells, plant pathogens have evolved various strategies to deregulate host immune responses [10, 11]. It has been posited that animal pathogens may not require any effector due to the non-intimate relationships between pathogens and animal cells [11, 26]. In this study, we report that the genomes of animal pathogenic fungi encode different numbers of LysM domain-containing proteins. Our functional studies revealed that two of 12 LysM proteins, i.e., Blys2 and Blys5, are required for the full virulence of the insect pathogen *B*. *bassiana*. These proteins can be secreted and function as *bona fide* effectors by targeting fungal cell wall chitin to deregulate insect host immunities. Of particular interest, the gene-rescue experiment with the Slp1 effector from the rice blast fungus *M*. *oryzae* could restore the virulence defect of Δ*Blys2* and Δ*Blys5* against insect hosts. The results of this study confirm that animal pathogens can employ a similar strategy to that used by plant pathogenic fungi for the effector-mediated evasion of host immune defenses to facilitate fungal infection.

LysM proteins are widely distributed in different organisms, from bacteria to fungi to plants [14]. Protein length diversity, domain number and sequence variations of LysM proteins are observed in different fungi, even between closely related fungal species. For example, 12 LysM proteins are present in the genome of *B*. *bassiana* but 14 in *B*. *brongniartii*. The inter- and intra-specific functional diversities of LysM proteins are still unknown. We found that the 12 *Blys* genes were differentially regulated by *B*. *bassiana*, and only the *Blys2* and *Blys5* genes that were activated in insect hemocoels were virulence factors. A recent proteomic investigation of the cell wall proteins of *B*. *bassiana* indicated that Blys8 could be identified from the hyphal bodies isolated from insect hemocoels [7]. We found that deletion of *Blys8* did not impair fungal virulence. Considering that *B*. *bassiana* is also a plant endophyte [32], other Blys proteins may be involved in fungal interactions with plants that remain to be determined.

Variations in LysM protein numbers were also observed among different strains of the plant pathogen *V*. *dahliae*. Moreover, a lineage-specific LysM effector but not the core LysM proteins (those present in all strains) was found to contribute to the virulence of the strain to alternative plant hosts [20]. This finding suggests that the LysM effector may play a role in influencing fungal host ranges. For insect pathogenic *Metarhizium* species, 13 LysM proteins are present in the genome of the generalist pathogen *M*. *robertsii*, whereas only four are present in the specialist species *M*. *album* and *M*. *acridum* [27]. This variation further suggests that the LysM protein may be connected with fungal lifestyles, including host specificity. In addition to suppress chitin-triggered immunity, the LysM proteins ChElp1 and ChElp2 of *C*. *higginsianum* are also required for the appressorium-mediated penetration of plant cells [19]. Interestingly, the LysM protein Tal6 from the mycoparasite *Trichoderma atroviride* could specifically inhibit the germination of the spores of *Trichoderma* species but not other fungi [33]. Thus, the exact function(s) of LysM protein remains to be determined in a species-specific manner.

Varied numbers of LysM domains (from 1–7) are present in different proteins. Except for the lineage-specific clustering pattern of the LysM1 and LysM2 domains retrieved from those proteins containing additional ChtBD1 and Glyco_18 domains, most LysM domains are clustered independent of their positions within the parental proteins (S3 Fig). Functional diversities of these variations are unclear. Structural analysis of the effector Ecp6 (containing three LysM domains) indicated that LysM1 and LysM3 can tightly form an intrachain dimer to mediate chitin binding with ultra-high affinity whereas the remaining LysM2 binds chitin with low affinity [21]. The effectors Slp1, ChElp1, ChElp2 and Vd2LysM each have two LysM domains, whereas Mg1LysM only contains a single LysM [17, 19, 20]. The formation of LysM dimers between the compartmented domains is therefore not applicable to these proteins that all can bind chitin but not chitosan, xylan and cellulose. We performed truncation studies of Blys2 and found that the five truncated forms retained the ability to bind chitin. However, the Blys2^D2-5^ form could additionally bind chitosan and cellulose with low affinity, whereas the losses of LysMs 1–2 (i.e., Blys2^D3-5^) or LysMs 1–3 (i.e., Blys2^D4-5^) reduced the mutant proteins’ chitin-binding affinity (Fig 3B). These results would suggest that the LysM1 of Blys2 might affect the protein binding specificity whereas the LysM2 and LysM3 domains might determine the protein’s chitin-binding affinity. Unlike Blys2, Blys5 with two LysM domains could additionally bind chitosan and cellulose. It has been reported that the Tal6 of *Trichoderma*, with seven LysM domains, could bind chitosan but not the chitin and cell walls of various fungal species. However, the truncated form of Tal6 that contains the last four LysM domains could bind colloidal chitin but not chitin powder and chitin flakes [33]. Thus, both the sequence and combination of LysM domains jointly determine the carbohydrate-binding specificity and/or affinity of LysM proteins.

To suppress the chitin-induced immune responses in plants, the LysM effectors upregulated by fungal pathogens can function as a competitive inhibitor of plant chitin receptors, a scavenger of chitin oligomers and/or a protective coat to shield fungal cells from the hydrolytic activity of plant chitinases [11, 12]. In contrast to the identification of LysM kinase receptors in plants [34], chitin receptor has not been identified in insects. Nevertheless, chitin oligomers could activate the expression of antimicrobial peptides in insects [35]. It has also been reported that the alternative chitinases encoded in insects might play a role in immune defense against chitin-containing pathogens [36]. In this respect, it is not surprising that entomopathogenic fungi have evolved similar strategies to suppress chitin-induced immunity in insects. Similar to the finding that extracellular ChElp2 is localized in fungal cell walls of *C*. *higginsianum* at the biotrophic interface [19], secreted Blys2 can target the cell walls of *B*. *bassiana*. In addition, we found that the loss of *Blys*2 could lead to the delay of fungal cell escape from hemocyte encapsulation and the upregulation of antifungal peptide gene in insects. Deletion of *Blys5* could also result in a higher level of activation of antifungal gene expression in insects when compared to the infection by the WT strain of *B*. *bassiana*. In contrast to Blys2, Blys5 can additionally protect fungal hyphae against chitinase degradation. Taken together, these results suggest therefore that the Blys2 effector could coat and protect the cell walls of insect pathogens from host cell recognition while Blys5 could additionally shield fungal cells from the hydrolysis of insect chitinases, the non-redundant functions of these two proteins in evasion of insect immune defenses.

The study of Slp1 in *M*. *oryzae* revealed that the effector is a competitive inhibitor of the rice receptor CEPiB to suppress chitin-induced immune responses in rice cells [17]. Since the virulence defects of Δ*Blys2* and Δ*Blys5* could be heterologously restored by Slp1, it cannot be ruled out that both Blys2 and Blys5 may also be able to outcompete the chitin receptor of insects, if any, to deregulate host immunities. Cell wall re-modifications occur during fungal invasion of the insect body cavity [3]. Consistent with a previous observation [31], we found that the chitin content of cell walls was reduced when the fungus was growing in insect hemocoels (Fig 4). This result could help explain why the overexpression of Blys2 and Slp1 did not lead to an obvious increase of fungal virulence that might be due to the saturation of the chitin substrate. As indicated above, Blys5 can additionally bind chitosan, a biopolymer that is rich in insects and has an immediate antifungal activity [37]. Thus, besides its shield effect against chitinases, Blys5 may additionally contribute to the detoxification of insect chitosan to facilitate fungal growth in insects.

In conclusion, we report the presence of diverse LysM proteins in animal pathogenic fungi and reveal that, similar to the LysM effectors of plant pathogenic fungi, the extracellular LysM proteins are virulence factors of the insect pathogen *B*. *bassiana*. Alternative proteins can bind chitin, coat fungal cell walls, deregulate insect immunities and/or protect fungal cells from host chitinase damage to facilitate fungal infection. Intriguingly, we found that the highly divergent plant pathogen effector Slp1 could restore the virulence defect of the *Blys2* and *Blys5* deletion mutants. The results of this study not only expand our knowledge of LysM protein evolution and functional diversification/similarity but also establish that the employment of effectors to evade host immunities similarly occurs during fungal interactions with animal hosts.

## Materials and methods

### Fungal strains and growth conditions

The WT and mutants of the *B*. *bassiana* strain ARSEF 2860 were routinely cultured on PDA (BD Difco) at 25°C for two weeks for conidial spore isolation. The rice blast fungus *M*. *oryzae* strain 70–15 was used to amplify the *Slp1* gene. For liquid incubation, fungi were grown in Sabouraud dextrose broth (SDB, BD Difco) at 25°C in a rotatory shaker. Conidium suspensions were prepared in 0.05% (v/v) Tween-20 and filtered through four layers of sterile lens-cleaning tissues to remove hyphal fragments for different experiments. The WT and mutant strains were also grown on PDA amended with H_2_O_2_ (3 mM) and Calcofluor White (200 μg/ml) at 25°C for different times [38]. The cultures were additionally incubated at 37°C to compare the stress responses between the WT and mutant strains.

### Bioinformatic and phylogenetic analyses

The proteins containing LysM domain(s) were retrieved from the genomes of 13 insect pathogenic fungi (S1 Table), 13 plant pathogenic fungi, and nine mammalian pathogenic fungi (S2 Table) using the program HMMER 3.1 (http://hmmer.org/). The obtained sequences were manually curated for further analysis of signal peptide, cysteine-residue ratio, transmembrane feature and length of the LysM domain proteins. A phylogenetic tree was constructed for the LysM domain-containing proteins retrieved from the genomes of the 13 insect pathogenic fungi mentioned above and the three plant pathogenic fungi that have been investigated and genome sequenced: *M*. *oryzae*, *F*. *oxysporum* and *Zymoseptoria tritici*. Thus, the full sequences of each protein were aligned using the program MUSCLE 3.8.31 [39], and a maximum likelihood tree was constructed using RAxML (ver. 3.1) using a WAG model and a bootstrap test of 1,000 replicates. The conserved sites at LysM domains extracted from the Pfam PF01476 of proteins from bacteria (8,738 proteins), proteins from insect pathogens (133) and plant pathogens (96) were searched and plotted using the program GLAM2 [40]. The LysM domain sequences extracted from the selected proteins (S1 and S2 Tables) were also used for neighbor-joining of phylogenetic analysis using MEGA (ver. 7.0) [41] with a bootstrap test of 1,000 replicates.

### Quantitative gene expression analysis

Twelve LysM domain-containing protein genes are encoded by *B*. *bassiana* [30]. To examine the expression of these genes, total RNA was extracted from the mycelia or blastospores harvested from SDB and the conidial spores or hyphae from the PDA plates. To determine gene expression during fungal *in vivo* infection, the last instar larvae of wax moth (*G*. *mellonella*) were individually injected with 10 μl of spore suspension (10^7^ spores/ml) for 60 hrs. Insect hemolymph was collected on ice, and fungal hyphal bodies were harvested by gradient centrifugation using Centricoll (Sigma-Aldrich) [30]. Each RNA sample was converted to cDNA using an AffinityScript multiple-temperature cDNA synthesis kit (Toyobo). qRT-PCR analysis was performed using a SYBR Premix Ex *Taq* kit (Takara) containing the primer pairs for different genes (S3 Table) on an ABI Prism 7000 system (Applied Biosystems). The β-tubulin gene (BBA_07018) of *B*. *bassiana* was amplified as an internal control. To determine insect antifungal gallerimycin gene expression, the last instar wax moth larvae were individually injected with the spore suspensions of WT and mutants for 36 hrs. Insect fat bodies were then dissected on ice and collected for RNA extraction to quantify the expression of the antifungal gene [9].

### Gene deletion, heterologous complementation and genetic transformation

Based on the RT-PCR analysis, the genes *Blys2*, *Blys4*, *Blys5*, *Blys6*, *Blys7* and *Blys8* were found to be transcribed by the fungus during growth in insect hemocoels. Targeted gene deletion of these sixe genes was individually performed by homologous recombination via *Agrobacterium*-mediated fungal transformation as previously described [42]. Briefly, the 5′- and 3′-flanking sequences were amplified using genomic DNA as a template with the appropriate primer pairs (S3 Table). The products were purified, digested with restriction enzymes and then inserted into the corresponding sites of the binary vector pDHt-bar (conferring resistance against ammonium glufosinate) to generate different plasmids (S4 Table) for transformation of the WT strain. In addition, the LysM effector Slp1 gene of *M*. *oryzae* [17] was amplified with the primers Slp1F/Slp1R and placed under the control of a constitutive *GpdA* gene (BBA_05480) promoter, and the cassette was integrated into the binary vector pDHt-ben (conferring resistance against benomyl) to transform the deletion mutants Δ*Blys2* and Δ*Blys5* for heterologous complementation. The *Slp1*-gene cassette was also cloned into the plasmid pDHt-bar to transform the WT strain for constitutive expression. A laccase gene (BBA_08183) promoter was also used to control *Blys2* to transform the WT because this laccase gene was highly transcribed by the fungus during growth in insect hemocoels [30]. The obtained mutants (S4 Table) were verified by PCR and RT-PCR analyses using the corresponding primer pairs (S3 Table).

### Protein localization and Western blotting assays

To determine protein localization, Blys2 and Slp1 were individually fused in frame at the C-termini with a GFP protein, and the cassettes were placed under the control of the *GpdA* gene promoter. The binary vectors were used to transform the WT strain of *B*. *bassiana*. The truncated Blys2 without signal peptide region (Blys2-SP) was also fused with the GFP protein. The WT and obtained mutants were grown under various nutrient conditions, and the fungal cells were harvested for microscopic observations. For chitin staining, fungal cells were incubated with 0.01% Calcofluor White (Sigma-Aldrich) buffered in 10% KOH for 1 min and rinsed twice with phosphate buffered saline (PBS, pH 8.0) before examination using a confocal microscopy (TCS SP8, Leica). To determine the secretion of Blys2, the obtained mutants engineered with GFP-fused Blys2 with and without SP regions were grown in SDB medium in a rotatory shaker at 220 rpm for three days. The cultures were filtered through filter paper, and the filtrates were further treated with a syringe filter unit (GP/SLGP033RS, Millipore) to remove fungal cells. Extracellular proteins were concentrated by precipitation with ammonium sulfate. The samples were centrifuged at 15,000× g for five minutes, and the proteins were reconstituted in Tris-HCl buffer (pH 8.0) and kept at 4°C. Mycelial samples were washed twice with sterile water and homogenized for total protein extraction. Extracellular and intracellular protein samples were separated using a 12% SDS-PAGE gel, and the Western blotting analysis was performed using the anti-GFP antibody (Abcam, China).

### Extraction of chitin from fungal cell walls

To determine the binding feature of Blys2 and Blys5 to cell wall chitins, conidia of *B*. *bassiana* were harvested from the two-week old of PDA plates, and suspended in 1 ml of 0.05% (v/v) Tween-20. The suspension was filtered through two layers of sterile filter paper to remove fungal hyphae. The conidia were collected by centrifugation, washed twice with distilled water and then resuspended in 5% (w/v) KOH for boiling for 30 min. After cooling down, cell wall chitin sample was collected by centrifugation at 15,000× g for 3 min, and the pellets were then washed with distilled water for three times, and resuspended in the solution of 40% H_2_O_2_ and glacial acetic acid (1:1) for boiling in water for 45 min [43]. Chitin was collected by low speed of centrifugation. The obtained pellets were washed for three times and resuspended in PBS (pH 8.0) for experiments.

### Protein expression and polysaccharide binding assays

To determine the effect of LysM domain number on chitin binding, we performed Blys2 protein truncations and polysaccharide binding assays. Thus, the primer pairs BF1/BR2, BF1/BR4, BF2/BR5, BF3/BR5 and BF4/BR5 (S3 Table) were used to amplify the regions containing the LysM domains 1–2, 1–4, 2–5, 3–5 and 4–5 of Blys2, respectively. The cDNA of Blys5 was amplified with the primers B5F and B5R. The purified products were integrated into the expression vector pET28b containing the His ×6 tag at the C-terminus using the Gateway clone system (Invitrogen). The plasmids were individually transformed into the BL21 (DE3) strain of *E*. *coli* for expression. For binding assays, the purified proteins (at a final concentration of 20 μg/ml each) were individually incubated with 3 μg of fungal chitin isolated above, chitin beads (Bioleaf, China), chitosan (Sigma-Aldrich) and cellulose (Sigma-Aldrich) in a total volume of 800 μl of water for 30 min with gentle rotating at room temperature. The samples were centrifuged at 15,000× g for 5 min [15]. The supernatants were collected, and the pellets were washed with PBS for three times. A SDS-PAGE analysis was conducted to detect the proteins in the supernatants and those bound to polysaccharides.

### Cell wall protection assay

To determine the potential effect of Blys2 and Blys5 on protecting fungal cell walls against chitinase, the WT and WT::*Slp1* spores were germinated in SDB (at a final concentration of ca. 1 × 10^5^ spores /ml) for 16 hrs. The germlings were harvested by centrifugation and washed twice with the 0.1 M potassium phosphate buffer containing 0.7 M KCl and 5 mM MgSO_4_. The WT germlings were pre-incubated with either Blys2 or Blys5 (10 μg) for 2 hrs, and additional WT (mock control) and the WT::*Slp1* germlings were treated with the buffer for 2 hrs. The samples were washed twice with the buffer and then treated with the buffered chitinase (Sigma-Aldrich; 0.2%, w/v) mixtures containing β-glucuronidase (Sigma-Aldrich; 0.4%, w/v), cellulase (Sigma-Aldrich; 0.4%, w/v) and lysozyme (Yeasen; 0.4%, w/v) [5] for 2 hrs at 30°C under gentle shaking. The rate of protoplast formation was estimated for each sample by examining 50 microscopic fields.

### Insect bioassays

Insect bioassays were conducted against the newly emerged last instar larvae of wax moth *G*. *mellonella*. Conidial suspensions were prepared for both topical infection (1 × 10^7^ conidia/ml) and injection (1 × 10^6^ conidia/ml) assays. Each treatment had three replicates with 15 insects each, and the experiments were repeated twice. For injection assays, spore suspensions (10 μl each) were injected into the base of the second proleg of the insects. Additional insects were injected and bled at various times to examine insect cellular immune response and fungal developments within the insect hemocoels. Insect mortality was recorded every 12 h after the treatments, and the LT_50_ values were calculated for each strain by Kaplan-Meier analysis. The differences were estimated between the WT and each mutant by the Log-rank test with the program SPSS (ver. 19) [44].

## Supporting information

S1 Table LysM domain-containing proteins identified from 13 species of entomopathogenic fungi. (XLSX)

S2 Table LysM domain-containing proteins identified from the selected plant and mammalian pathogenic fungi. (XLSX)

S3 Table Primers used in this study. (DOCX)

S4 Table Vectors constructed and mutants obtained in this study. (DOCX)

S1 Fig Characteristic analysis of fungal LysM proteins. Based on the parameters of the protein length and cysteine ratio of 282 proteins retrieved from the insect, mammalian and pathogenic fungi (S1 and S2 Tables). The examined proteins can be clearly divided into two groups. Fewer proteins from plant pathogens are clustered in the g2 group when compared to those from the insect and mammalian pathogenic fungi.
(TIF)

S2 Fig Phylogenetic and structure analysis of fungal LysM proteins. The protein sequences were aligned with MUSCLE and a maximum likelihood tree was generated using a WAG model with the bootstrap test of 1,000 replicates. Each protein is aligned with its secondary structure that contains different domains as indicated. The LysM proteins from *B*. *bassiana* are highlighted in red.
(TIF)

S3 Fig Phylogenetic analysis of the LysM domain sequences retrieved from different fungal proteins. Individual LysM domain was retrieved from each examined protein and tagged with a number indicating its sequential position within the parental protein. The sequences were aligned and a neighbor-joining tree was generated with the bootstrap test of 1,000 replicates. P, the specific lineage of the LysM domains from the proteins of plant pathogens; C1-C4, the lineages clustered in association with LysM domain positions showing in the figure. The LysM domains from the proteins of *B*. *bassiana* are highlighted in bold. The branches in green show the LysM domains retrieved from the proteins of plant pathogenic fungi.
(TIF)

S4 Fig Conservation analysis of the LysM domain sequences from different organisms. **A**. Sequence consensus of the LysM domains extracted from 8,738 bacterial proteins catalogued at the Pfam family PF01476. **B**. Sequence consensus of the LysM domains extracted from the proteins of the selected plant pathogenic fungi (S2 Table) that do not contain a cysteine-residue at the second residue position. **C**. Sequence consensus of the LysM domains extracted from the proteins of the selected plant pathogenic fungi (S2 Table) that contain a cysteine-residue at the second residue position. **D**. Sequence consensus of the LysM domains extracted from the proteins of the selected insect pathogenic fungi (S1 Table).
(TIF)

S5 Fig Verification of gene deletions and phenotypic characterization of gene deletion mutants. **A**. RT-PCR verification of gene deletions. The WT and Δ*Blys2* cultures harvested from SDB for three days; the WT, Δ*Blys4*, Δ*Blys5*, Δ*Blys6* and Δ*Blys8* harvested from PDA for three days, and the WT and Δ*Blys7* harvested from PDA for ten days were used for RNA extraction, respectively. *Tub*, a β-tubulin gene used as a reference. **B**. Characterization and comparison of the WT and mutant growth on different media. Spore suspensions of the WT and mutants were prepared (2×10^7^ spores/ml), diluted 10 times in serial, and 10 μl of each suspension were inoculated on PDA for three days, PDA plus 3 mM H_2_O_2_ for four days, and PDA plus Calcofluor White (CW, 200 μg/ml) for three days.
(TIF)

S6 Fig Kinetics of insect survivals after the treatments with the WT and gene deletion mutants. **A**. Survival of the wax moth larvae following the injection with the spores (1 × 10^6^ conidia/ml; 10 μl each) of the WT and gene deletion mutants. Control insects were injected with 10 μl of 0.05% Tween 20. **B**. Survival of the wax moth larvae following the topical infection with the spore suspensions (1 × 10^7^ conidia/ml) of the WT and gene deletion mutants. Control insects were treated with 0.05% Tween 20.
(TIF)

S7 Fig Kinetics of insect survivals after the treatments with the WT and different mutants. **A**. Survival of the wax moth larvae following the injection with the spores (1 × 10^6^ conidia/ml; 10 μl each) of the WT and *Blys2*-related mutants. Control insects were injected with 10 μl of 0.05% Tween 20. **B**. Survival of the wax moth larvae following the topical infection with the spore suspensions (1 × 10^7^ conidia/ml) of the WT and *Blys2*-related mutants. Control insects were treated with 0.05% Tween 20. **C**. Survival of the wax moth larvae following the injection with the spores of the WT, WT::*Slp1* and *Blys5*-related mutants. Control insects were injected with 10 μl of 0.05% Tween 20.
(TIF)

S8 Fig Conservation and phylogeny analysis of the LysM domains retrieved from the selected proteins. **A**. Alignment of individual LysM domains from Blys2, Blys5, Slp1 and Ecp6. **B**. Phylogram with distance indicator showing the relatedness of the LysM domains from four proteins. The tree was inferred using the Neighbor-Joining method with the bootstrap test of 1,000 replicates. The percentage of > 50% replicate supports in which the associated taxa clustered together are shown next to the branches.
(TIF)
