# Supplementary material for: Divergent LysM effectors contribute to the virulence of Beauveria bassiana by evasion of insect immune defenses
Source: PLoS Pathog. 2017 Sep 5;13(9):e1006604. doi: 10.1371/journal.ppat.1006604 (PMC5600412; doi:10.1371/journal.ppat.1006604)
Supplement: S3 Table — (DOCX) [file ppat.1006604.s003.docx]

**S3 Table. Primers used in this study.**

| **Primer** | **Sequence (5'-3')** |
| --- | --- |
| RT-PCR or qRT-PCR uses for detection of gene expression | |
| Blys1F | CGTACCAACATCCCAGCAAG |
| Blys1R | CGTACCAACATCCCAGCAAG |
| Blys2F | CACCACTCTCACTACCGCTG |
| Blys2R | ACCCAAAGACCAGTGCAGTC |
| Blys3F | TGTTCTGGTCGCTACTCTGG |
| Blys3R | GAAACGCCGATACAGACGTC |
| Blys4F | GTGAAGACGACGATGCACAG |
| Blys4R | AGGCAGATCTGTGAACCGAT |
| Blys5F | CTCCGTCATTTCTGCCGTTT |
| Blys5R | CAGTAGTACCAGCCACCGAG |
| Blys6F | TGGTACGAGATTCAGACGGG |
| Blys6R | AGCCAAGGATTCCACTCGAT |
| Blys7F | CACGGCCACTTTCCAATCAT |
| Blys7R | AAATCGGACAGGTCGAGGTT |
| Blys8F | GGAAAGGACTGCTCAGGACT |
| Blys8R | TAAACTTCCACCCCTCCGAC |
| Blys9F | CCTATCCTGACTGTCCCGTC |
| Blys9R | GTTGTTTGCTCACCCGTCAT |
| Blys10F | AGTCAGGCATCAACAGCCTC |
| Blys10R | CCTACCTCGAACTCGGCATC |
| Blys11F | CGCAGCGCTGTCAATCTAAC |
| Blys11R | CTCGGCGTCTGAGATGTACC |
| Blys12F | GAGAGGCCATATACGGTGGC |
| Blys12R | TTGGCAGCGAGTCCATTGAT |
| Tub F | CAGGGCTTTCAGATTACGC |
| Tub R | CGACGGTGAAGAAATGGAG |
| Gene deletions | |
| Blys2Up F | GTACCGGGCCCCCCCAAAGTTGCAATGCAGCCTGG |
| Blys2Up R | TACCGTCGACCTCGACCTGGGTAGAACTGGGCTTG |
| Blys2Down F | CACCGCGGTGGAGCACTCCACAAGAGCACCACTG |
| Blys2Down R | AGGGAACAAAAGCTGATGCGTGCAGCAGTAACAG |
| Blys4Up F | GTACCGGGCCCCCCCACCAAAGAGAGCAGAG |
| Blys4Up R | TACCGTCGACCTCGATACATGCAATGCTTCGA |
| Blys4Down F | CACCGCGGTGGAGCTGAGCAACAACATAGCC |
| Blys4Down R | AGGGAACAAAAGCTGGTCCCTATACTGGTTGC |
| Blys5Up F | GTACCGGGCCCCCCCGTTGCATGTCTCGTCA |
| Blys5Up R | TACCGTCGACCTCGATTGGTGGCGGATGAAAA |
| Blys5Down F | CACCGCGGTGGAGCTAAAATGGTGGGCTGCT |
| Blys5Down R | AGGGAACAAAAGCTGGAATCCCGTGCGAACA |
| Blys6Up F | GTACCGGGCCCCCCCCTACCTTGAATTCACG |
| Blys6Up R | TACCGTCGACCTCGAGTCTACGAGTAATCCC |
| Blys6Down F | CACCGCGGTGGAGCTTCTCCTTCGCCACAAT |
| Blys6Down R | AGGGAACAAAAGCTGGTCGTTCCGTGATCAAA |
| Blys7Up F | GTACCGGGCCCCCCCGTACTGAGATCGCTGGCTCC |
| Blys7Up R | TACCGTCGACCTCGATGGTTGCGTTTTCGCTCAAG |
| Blys7Down F | CACCGCGGTGGAGCTACAAGGTTCGGCTGTACTGG |
| Blys7Down R | AGGGAACAAAAGCTCGCGGGAAAAGCAAGTCAAA |
| Blys8Up F | GTACCGGGCCCCCCCGGAAGAGAGAGAGGAC |
| Blys8Up R | TACCGTCGACCTCGACCGAATTGAGATGTGA |
| Blys8Down F | CACCGCGGTGGAGCTGAAAGGGCATGGGTGT |
| Blys8Down R | AGGGAACAAAAGCTGGCTGCCACCAATAGAA |
| Gene complementation | |
| Slp1R | GGAATTCGTTCTTGCAGATGGGGATGT |
| Slp1F | CTTTTAATCAATAACAATGCAGTTCGCTACC |
| Blys2CutF | GTCTAGAATGAAGTGCAGCTACAAGTGGCGC |
| Blys2F | GTCTAGAATGACTCGATTTACTACCAC |
| Blys2R | GGAATTCGTGGTGGTGGTGGTGGTGGCCTTGGACACCGACGCA |
| Gene fusion | |
| GFP F | ATGGTGAGCAAGGGCGAGG |
| GFP R | GTCTAGATGGACGAGCTGTACAAGTAA |
| Lac F | TTGTACCAAGGCCACCATGACTCGATTTACTAC |
| Lac R | GGTGGCCTTGGTACAAGATGCT |
| GpdA F | GGAATTCCTTGCTTCAAAACAGCCCAA |
| GpdA R | GAGTGGTAGTAAATCGAGTCATTGTTATTGATTAAAAGGGTGA |
| Blys2 truncation and expression (underlined sequences are from the pET28b vector) | |
| BF1 | GTCGGGATCCGAATTCGTGGCGCGCTCACGCCGG |
| BF2 | GTCGGGATCCGAATTCGGACGGTGTTGCCAAGGAC |
| BF3 | GTCGGGATCCGAATTCGCCCAGTTCTACCCAGGCT |
| BF4 | GTCGGGATCCGAATTCGCAGGACGGCCTCACCGAC |
| BR2 | AGCTTGTCGACGGAGCTGGTCGGCGAGGTAGGAGT |
| BR4 | AGCTTGTCGACGGAGCTGCCCGGGTTGGCGGGCGT |
| BR5 | AGCTTGTCGACGGAGCTGCCTTGGACACCGACGCA |
| Blys5 expression (underlined sequences are from the pET28b vector) | |
| B5F | AAATGGGTCGGGATCCGATGAAGCTCTCCGTCA |
| B5R | GTCGACGGAGCTCGAATTTCAGGCTTTGACACAGA |
| Insect antifungal gallerimycin gene expression analysis | |
| Gal F | AAGATCGCTTTCATAGTCGCA |
| Gal R | CTCGTAAAATACACATCCGGG |
