# Supplementary material for: Divergent LysM effectors contribute to the virulence of Beauveria bassiana by evasion of insect immune defenses
Source: PLoS Pathog. 2017 Sep 5;13(9):e1006604. doi: 10.1371/journal.ppat.1006604 (PMC5600412; doi:10.1371/journal.ppat.1006604)
Supplement: S4 Table — (DOCX) [file ppat.1006604.s004.docx]

**S4 Table. Vectors constructed and mutants obtained in this study.**

| **Vectors** | **Notes** | **Obtained mutants** |
| --- | --- | --- |
| pDHt-Δ*Blys2* | *Blys2* deletion vector | Δ*Blys2* |
| pDHt-Δ*Blys4* | *Blys4* deletion vector | Δ*Blys4* |
| pDHt-Δ*Blys5* | *Blys5* deletion vector | Δ*Blys5* |
| pDHt-Δ*Blys6* | *Blys6* deletion vector | Δ*Blys6* |
| pDHt-Δ*Blys7* | *Blys7* deletion vector | Δ*Blys7* |
| pDHt-Δ*Blys8* | *Blys7* deletion vector | Δ*Blys8* |
| pDHt-lac-Blys2-bar | For overexpression in the WT strain | WT::*lp-Blys2* |
| pDHt-gpdA-Blys2-bar | For overexpression in the WT strain | WT::*gp-Blys2* |
| pDHt-gpdA-Blys2-GFP-bar | For overexpression in the WT strain | WT::*gp-Blys2-GFP* |
| pDHt-gpdA-Blys2Cut-GFP-bar | For overexpression in the WT strain | WT::*gp-Blys2-SP-GFP* |
| pDHt-gpdA-Slp1-ben | For complementation of Δ*Blys2* | Δ*Blys2*::*Slp1* |
|  | For complementation of Δ*Blys5* | Δ*Blys5*::*Slp1* |
| pDHt-gpdA-Slp1-GFP-bar | For overexpression in the WT strain | WT::*gp-Slp1-GFP* |
| pDHt-gpdA-GFP-bar | For overexpression in the WT strain | WT::*GFP* |
