# Supplementary figures and images for: Divergent LysM effectors contribute to the virulence of Beauveria bassiana by evasion of insect immune defenses
Source: PLoS Pathog. 2017 Sep 5;13(9):e1006604. doi: 10.1371/journal.ppat.1006604 (PMC5600412; doi:10.1371/journal.ppat.1006604)

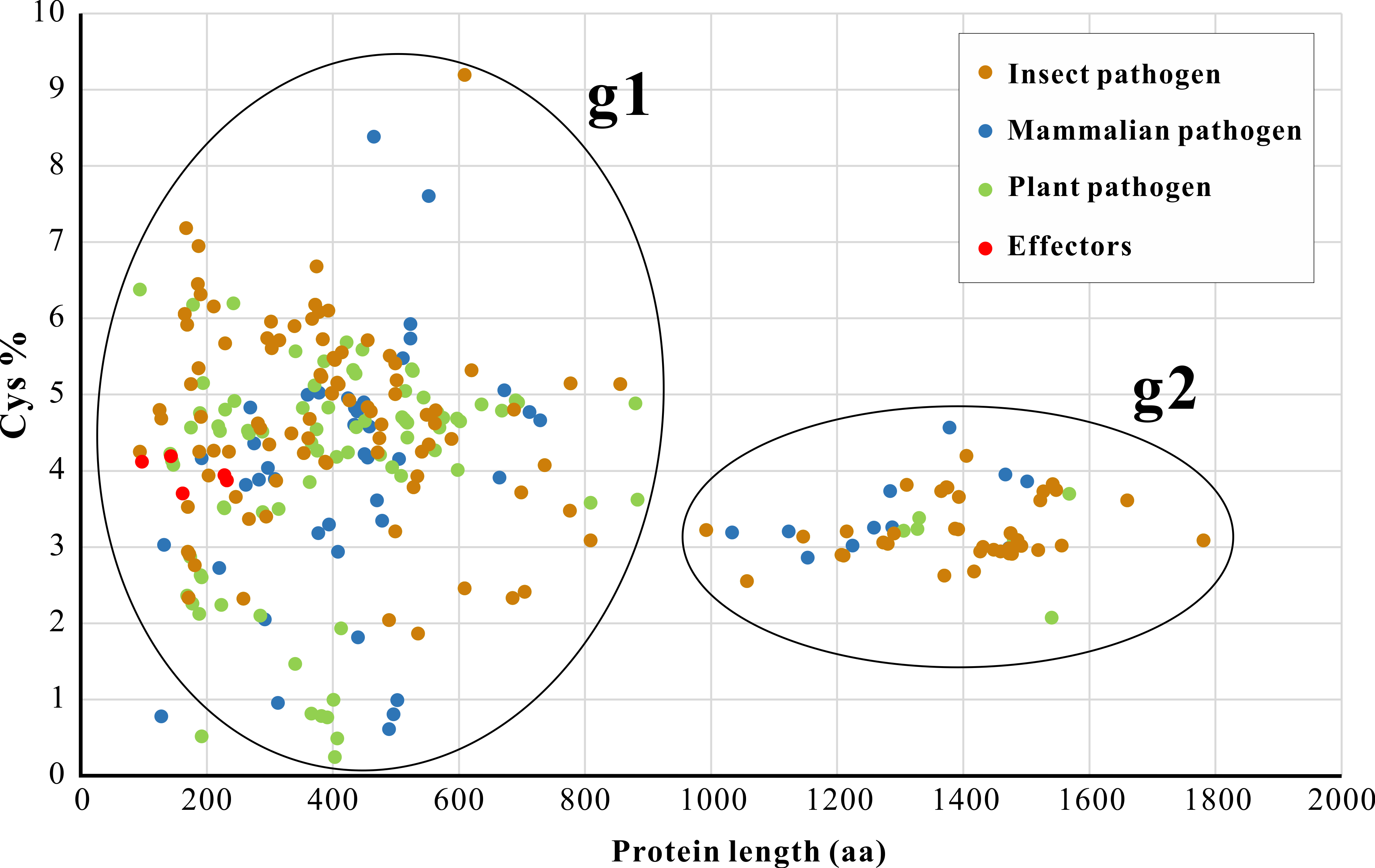

Supplement: S1 Fig — Based on the parameters of the protein length and cysteine ratio of 282 proteins retrieved from the insect, mammalian and pathogenic fungi (S1 and S2 Tables). The examined proteins can be clearly divided into two groups. Fewer proteins from plant pathogens are clustered in the g2 group when compared to those from the insect and mammalian pathogenic fungi. (TIF) [file ppat.1006604.s005.tif]

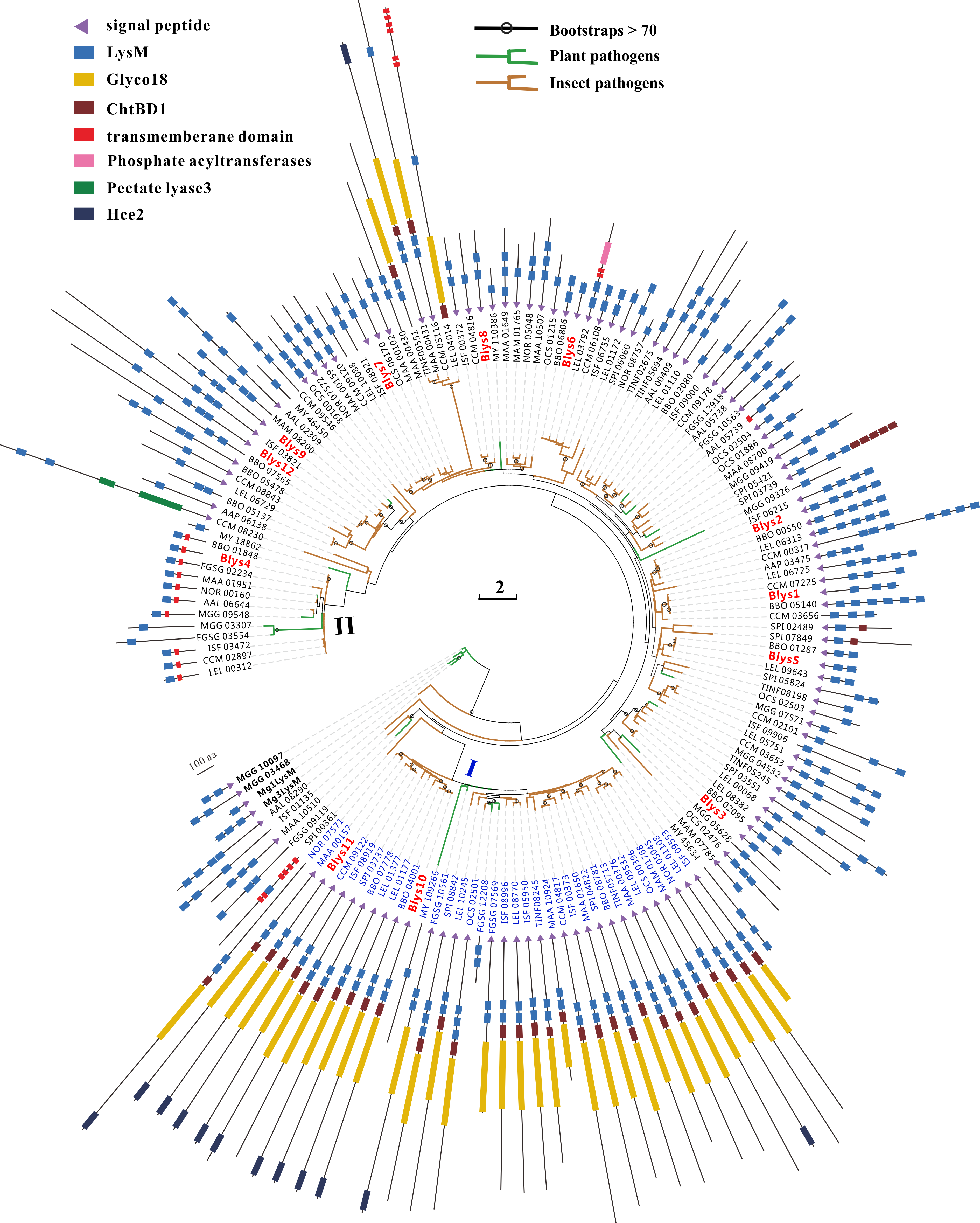

Supplement: S2 Fig — The protein sequences were aligned with MUSCLE and a maximum likelihood tree was generated using a WAG model with the bootstrap test of 1,000 replicates. Each protein is aligned with its secondary structure that contains different domains as indicated. The LysM proteins from B. bassiana are highlighted in red. (TIF) [file ppat.1006604.s006.tif]

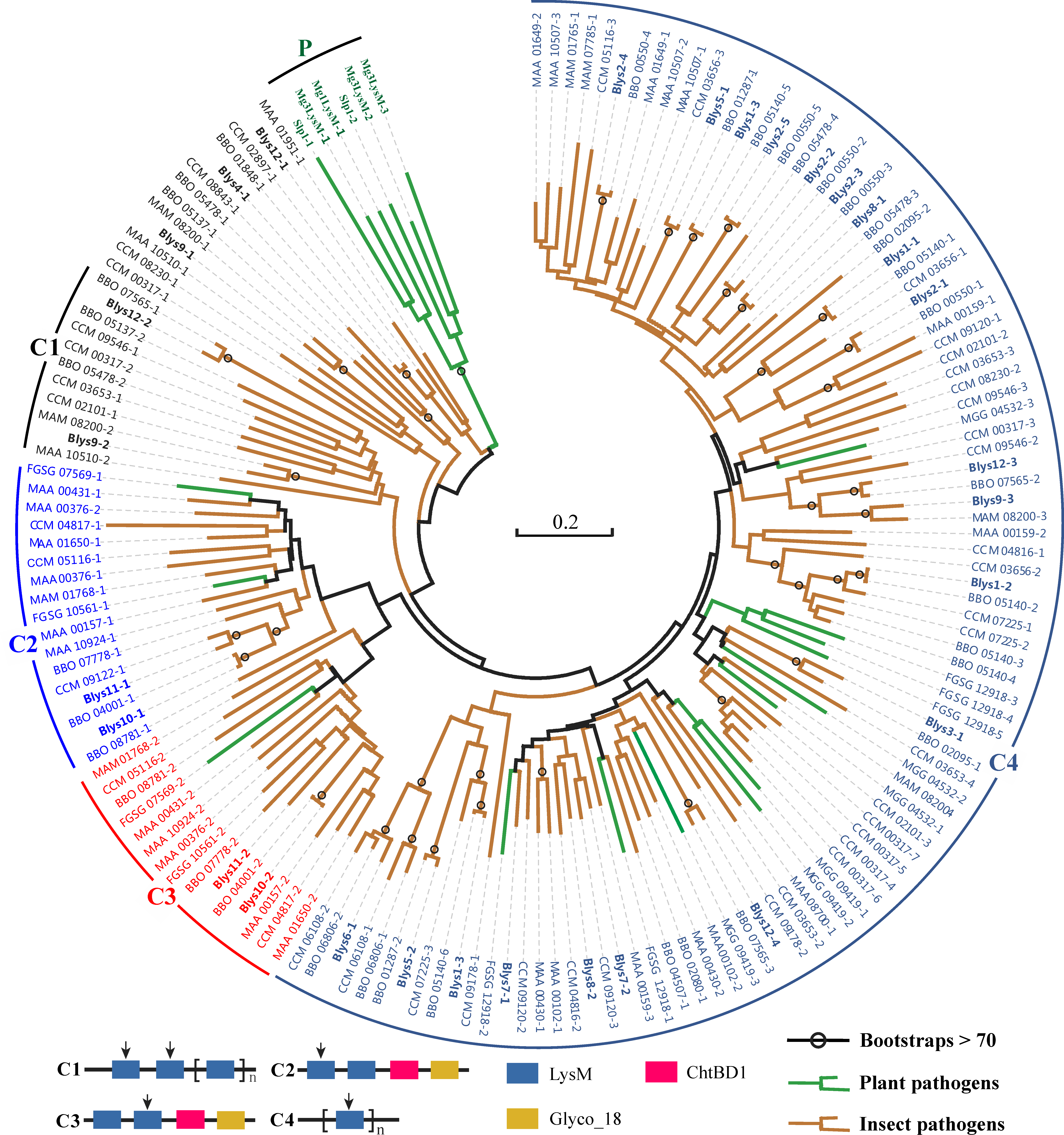

Supplement: S3 Fig — Individual LysM domain was retrieved from each examined protein and tagged with a number indicating its sequential position within the parental protein. The sequences were aligned and a neighbor-joining tree was generated with the bootstrap test of 1,000 replicates. P, the specific lineage of the LysM domains from the proteins of plant pathogens; C1-C4, the lineages clustered in association with LysM domain positions showing in the figure. The LysM domains from the proteins of B. bassiana are highlighted in bold. The branches in green show the LysM domains retrieved from the proteins of plant pathogenic fungi. (TIF) [file ppat.1006604.s007.tif]

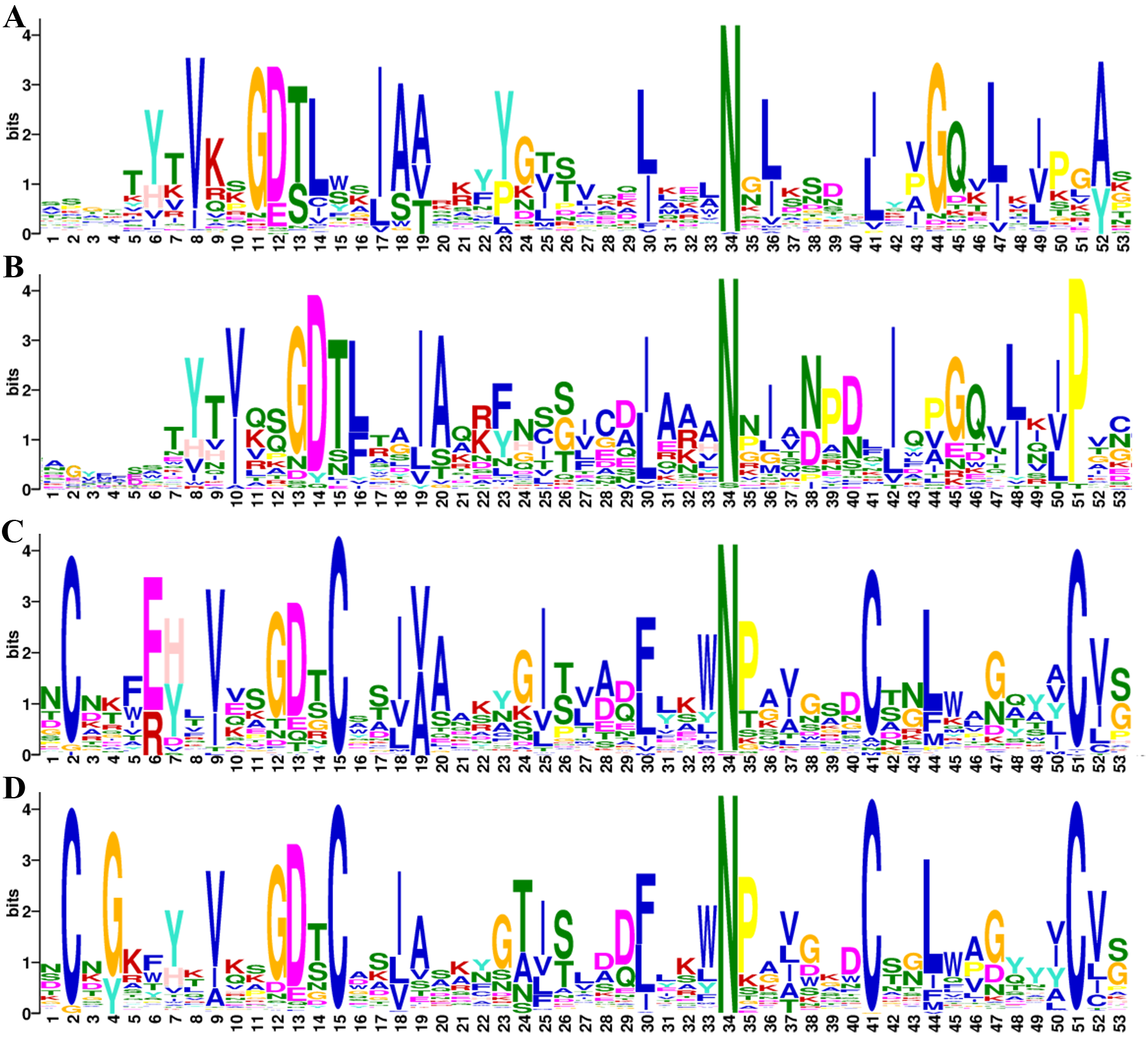

Supplement: S4 Fig — A. Sequence consensus of the LysM domains extracted from 8,738 bacterial proteins catalogued at the Pfam family PF01476. B. Sequence consensus of the LysM domains extracted from the proteins of the selected plant pathogenic fungi (S2 Table) that do not contain a cysteine-residue at the second residue position. C. Sequence consensus of the LysM domains extracted from the proteins of the selected plant pathogenic fungi (S2 Table) that contain a cysteine-residue at the second residue position. D. Sequence consensus of the LysM domains extracted from the proteins of the selected insect pathogenic fungi (S1 Table). (TIF) [file ppat.1006604.s008.tif]

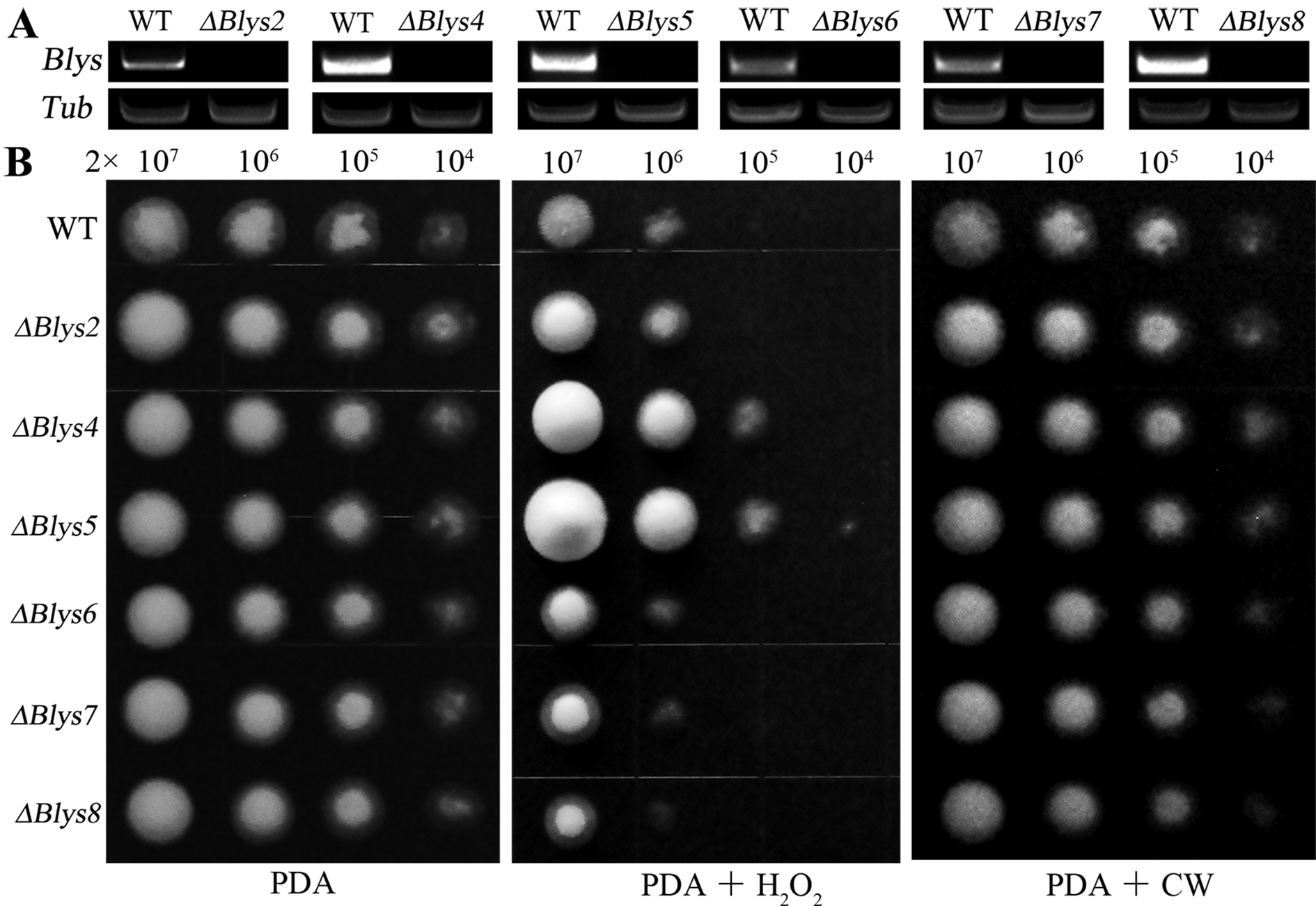

Supplement: S5 Fig — A. RT-PCR verification of gene deletions. The WT and ΔBlys2 cultures harvested from SDB for three days; the WT, ΔBlys4, ΔBlys5, ΔBlys6 and ΔBlys8 harvested from PDA for three days, and the WT and ΔBlys7 harvested from PDA for ten days were used for RNA extraction, respectively. Tub, a β-tubulin gene used as a reference. B. Characterization and comparison of the WT and mutant growth on different media. Spore suspensions of the WT and mutants were prepared (2×10^7^ spores/ml), diluted 10 times in serial, and 10 μl of each suspension were inoculated on PDA for three days, PDA plus 3 mM H2O2 for four days, and PDA plus Calcofluor White (CW, 200 μg/ml) for three days. (TIF) [file ppat.1006604.s009.tif]

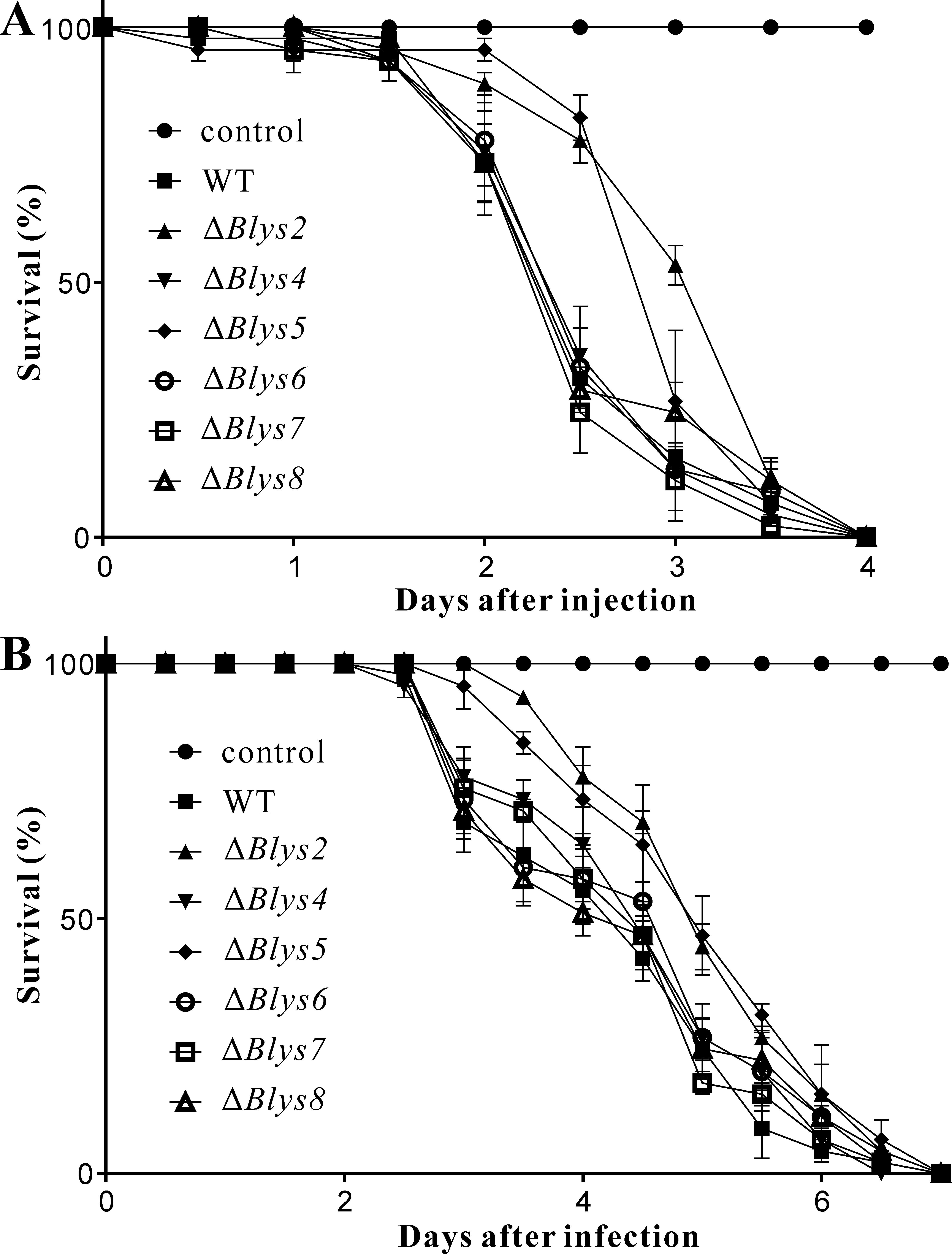

Supplement: S6 Fig — A. Survival of the wax moth larvae following the injection with the spores (1 × 10^6^ conidia/ml; 10 μl each) of the WT and gene deletion mutants. Control insects were injected with 10 μl of 0.05% Tween 20. B. Survival of the wax moth larvae following the topical infection with the spore suspensions (1 × 10^7^ conidia/ml) of the WT and gene deletion mutants. Control insects were treated with 0.05% Tween 20. (TIF) [file ppat.1006604.s010.tif]

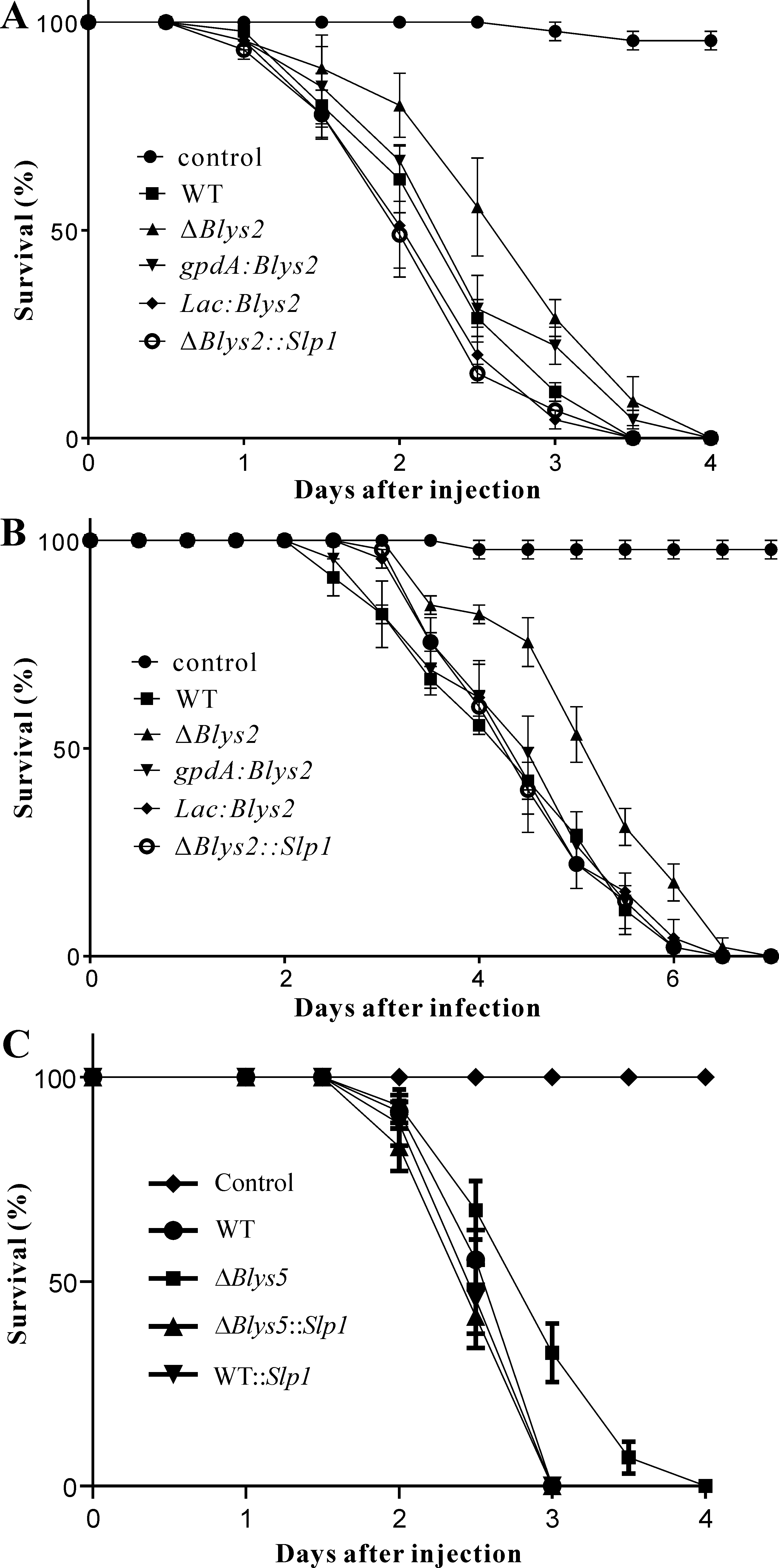

Supplement: S7 Fig — A. Survival of the wax moth larvae following the injection with the spores (1 × 10^6^ conidia/ml; 10 μl each) of the WT and Blys2-related mutants. Control insects were injected with 10 μl of 0.05% Tween 20. B. Survival of the wax moth larvae following the topical infection with the spore suspensions (1 × 10^7^ conidia/ml) of the WT and Blys2-related mutants. Control insects were treated with 0.05% Tween 20. C. Survival of the wax moth larvae following the injection with the spores of the WT, WT::Slp1 and Blys5-related mutants. Control insects were injected with 10 μl of 0.05% Tween 20. (TIF) [file ppat.1006604.s011.tif]

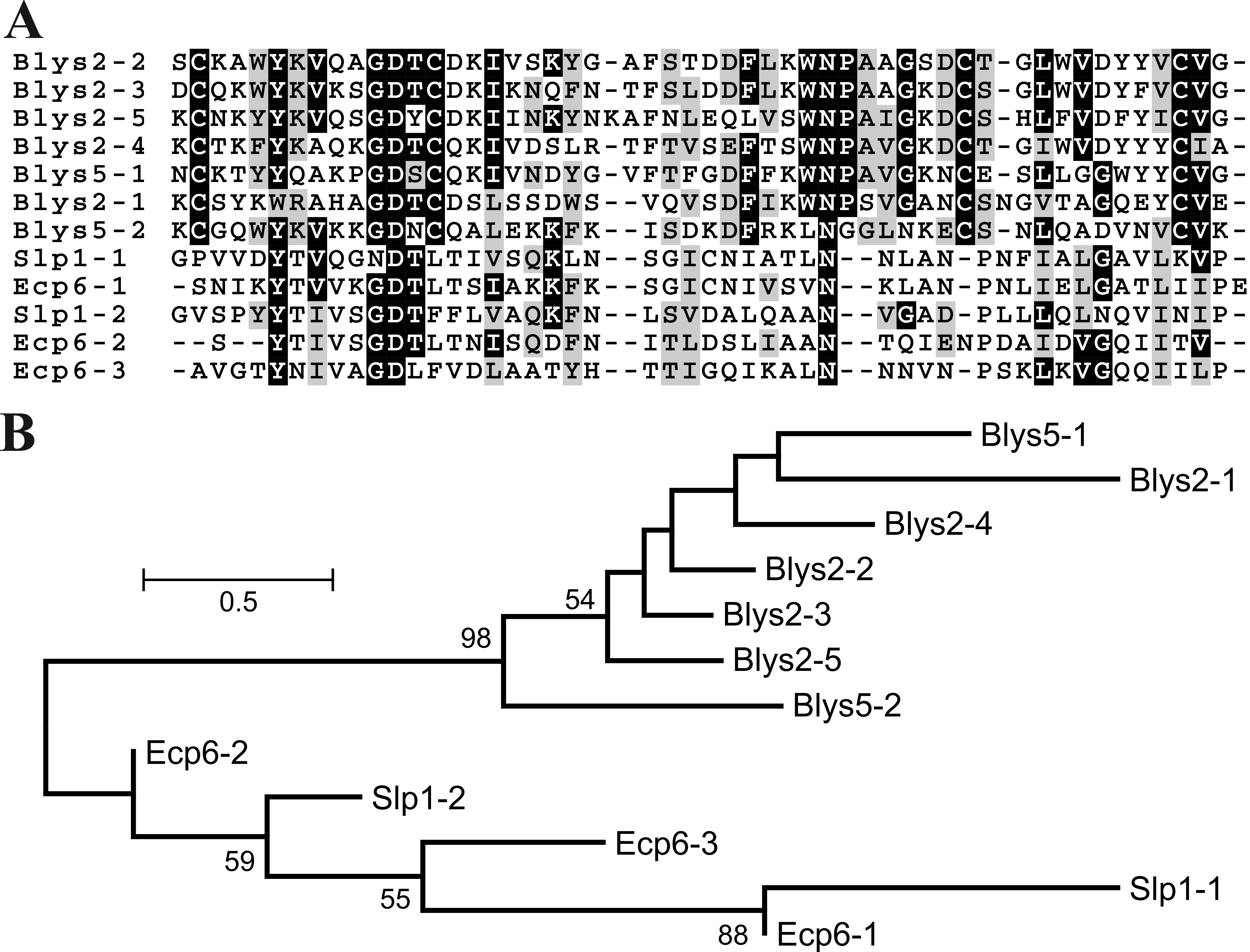

Supplement: S8 Fig — A. Alignment of individual LysM domains from Blys2, Blys5, Slp1 and Ecp6. B. Phylogram with distance indicator showing the relatedness of the LysM domains from four proteins. The tree was inferred using the Neighbor-Joining method with the bootstrap test of 1,000 replicates. The percentage of > 50% replicate supports in which the associated taxa clustered together are shown next to the branches. (TIF) [file ppat.1006604.s012.tif]
